# Nuclear export of BATF2 enhances colorectal cancer proliferation through binding to CRM1

**DOI:** 10.1002/ctm2.1260

**Published:** 2023-05-07

**Authors:** Jie Zhou, Zengjie Lei, Jianfang Chen, Shengbo Liao, Yanrong Chen, Chengxiang Liu, Shuo Huang, Liuli Li, Yan Zhang, Pei Wang, Yinghui Huang, Jianjun Li, Houjie Liang

**Affiliations:** ^1^ Department of Oncology and Southwest Cancer Center Southwest Hospital Army Medical University (Third Military Medical University) Chongqing China; ^2^ Department of Medical Oncology Affiliated Jinling Hospital Medical School of Nanjing University Nanjing China; ^3^ Department of Otolaryngology People's Hospital of Xishui County Guizhou China; ^4^ Department of Nephrology Key Laboratory for the Prevention and Treatment of Chronic Kidney Disease of Chongqing Chongqing Clinical Research Center of Kidney and Urology Diseases Xinqiao Hospital Army Medical University Chongqing China

**Keywords:** BATF2, colorectal cancer, CRM1, nuclear export

## Abstract

**Background:**

During the tumourigenesis and development of colorectal cancer (CRC), the inactivation of tumour suppressor genes is closely involved, although detailed molecular mechanisms remain elusive. Accumulating studies, including ours, have demonstrated that basic leucine zipper transcription factor ATF (activating transcription factor)‐like 2 (BATF2) is a capable tumour suppressor that localises in the nucleus. However, its different subcellular localisation, potential functions and underlying mechanisms are unclear.

**Methods:**

The translocation of BATF2 and its clinical relevance were detected using CRC samples, cell lines and xenograft nude mice. Candidate BATF2‐binding proteins were screened using co‐immunoprecipitation, quantitative label‐free liquid chromatography–tandem mass spectrometry proteomic analysis, Western blotting and immunofluorescence. Recombinant plasmids, point mutations and siRNAs were applied to clarify the binding sites between BATF2 and chromosome region maintenance 1 (CRM1).

**Results:**

The present study found that BATF2 was mainly localised in the cytoplasm, rather than nucleus, of CRC cells in vitro and in vivo, while cytoplasmic BATF2 expression was inversely correlated with the prognosis of CRC patients. Furthermore, we identified the nuclear export and subsequent ubiquitin‐mediated degradation of BATF2 in CRC cells. Mechanistically, a functional nuclear export sequence (any amino acid) was characterised in BATF2 protein, through which BATF2 bound to CRM1 and translocated out of nucleus, ultimately enhancing CRC growth via inducing activator protein 1 (AP‐1)/cyclin D1/phosphorylated retinoblastoma protein (pRb) signalling pathway. Additionally, nuclear export of BATF2 can be retarded by the mutation of NES in BATF2 or the knockdown of CRM1, whereas CRM1 expression was negatively associated with nuclear BATF2 expression and the prognosis of CRC patients.

**Conclusion:**

These findings revealed the biological effects and underlying mechanisms of cytoplasmic localisation of BATF2. Furthermore, suppressing nuclear export of BATF2 via mutating its NES region or inhibiting CRM1 expression may serve as a promising therapeutic strategy against CRC.

## Graphical abstract

1. Cytoplasmic BATF2 negatively correlates with the prognosis of CRC patients.

2. BATF2 interacts with CRM1 through its NES to translocate out of nucleus.

3.Nuclear export of BATF2 contributes to CRC proliferation via activating AP‐1/cyclin D1/pRb pathway.

4. Suppressing nuclear export of BATF2 via mutating its NES region or inhibiting CRM1 expression represses CRC growth.

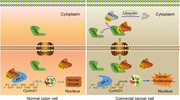

## 1 INTRODUCTION

Colorectal cancer (CRC) is one of the most common malignancies worldwide, with high morbidity and mortality.^1^ During the tumourigenesis and development of CRC, the inactivation of tumour suppressor genes (TSGs) is closely involved.^2^ It has been reported that multiple ways contribute to inactivating TSGs, including gene mutation, epigenetic modification, gene expression regulation and nucleus–cytoplasmic translocation (NCT).^3^, ^4^, ^5^ Of note, dysregulation of NCT leads to abnormal subcellular localisation of oncogenes and tumour suppressors, such as p53 and p27, eventually leading to the initiation and progression of CRC.^6^, ^7^, ^8^ Besides, the NCT of TSGs, such as adenomatous polyposis coli and p53, also plays a crucial role in tumour resistance to current treatments, including chemotherapies and targeted therapies, making it an attractive target for novel therapeutic strategies.^8^, ^9^, ^10^ However, the detailed molecular mechanisms of NCT remain elusive.

Basic leucine zipper (bZIP) transcription factor ATF‐like 2 (BATF2), also known as suppressor of activator protein 1 (AP‐1) regulated by interferon (SARI), was identified as a tumour suppressor, as well as a type I interferon‐inducible gene.^11^, ^12^ BATF2 belongs to AP‐1 family and interacts mainly with AP‐1 via its bZIP domain in the nucleus, leading to AP‐1 inhibition and cancer suppression.^11^, ^12^, ^13^, ^14^ Current studies demonstrate that BATF2 is expressed in the nucleus of normal cells, rather than in their malignant counterparts, while overexpression of BATF2 is able to suppress the proliferation, invasion and metastasis of cancer cells,^14^, ^15^ but not in normal cells,^11^ indicating the pivotal role of BATF2 in the field of cancer therapy.

To investigate the mechanism of the dysfunction of BATF2 in cancer, direct sequencing was performed, but no mutations in any of the three exons of *BATF2* gene were detected in hepatocellular carcinoma samples and cell lines.^16^ Therefore, subsequent studies, including ours, mainly focused on gene expression regulation through exploring the upstream transcriptional factors, microRNAs, circular RNAs and N6‐methyladenosine modification of BATF2.^12^, ^14^, ^15^, ^17^, ^18^, ^19^, ^20^ Our previous work also identified a transcriptional factor, glucocorticoid receptor, which could induce BATF2 expression in lymphoma cells.^12^ However, these studies have still not clarified the cause of the reduced tumour suppressive effect of BATF2. To the best of our knowledge, previous studies have consistently suggested that BATF2 is located in the nucleus,^11^, ^12^, ^13^, ^14^, ^15^, ^16^, ^17^, ^18^, ^21^ but its various subcellular localisations, as well as the potential biological effects and underlying molecular mechanisms, remain unknown.

In the present study, we reported for the first time that BATF2 was mainly localised in the cytoplasm of CRC cells, which was inversely correlated with the prognosis of CRC patients. Subsequent mechanism studies demonstrated that BATF2 interacted with chromosome region maintenance 1 (CRM1) through its nuclear export sequence (NES) region, which led to the nuclear export and ubiquitin‐mediated degradation of BATF2, eventually contributing to CRC proliferation via activating AP‐1/cyclin D1/phosphorylated retinoblastoma protein (pRb) signalling pathway. These findings collectively revealed the biological effects and underlying mechanisms of cytoplasmic localisation of BATF2, which may shed new light on exploring potential therapeutic targets for CRC patients.

## 2 METHODS

### 2.1 Human samples

Human CRC and pericarcinoma tissue microarrays from 184 patients were obtained from the National Human Genetic Resources Sharing Service Platform (Shanghai Outdo Biotechnology, No. YBM‐05‐02, Shanghai, China), and 30 cases of tumour tissues, including CRC, liver cancer, bile duct cancer, gastric cancer, kidney cancer, lung adenocarcinoma, lung adenosquamous carcinoma, lung squamous cell carcinoma, nasopharyngeal carcinoma and pancreatic adenocarcinoma, were obtained from the People's Hospital of Xishui County with approval from its ethics committee (No. 2021XRYLS1), and written informed consent was obtained from all patients. Tumours were staged blindly by three pathologists in the Department of Pathology of Southwest Hospital. The clinicopathological parameters of CRC patients, including gender, age, diagnosis and classification, are shown in Table S1.

### 2.2 Cell culture

The human normal colon epithelial cells (CCD 841 CoN) and CRC cells (HCT116) were purchased from American Type Culture Collection (Manassas, VA, USA) and cultured in high‐glucose Dulbecco's modified Eagle medium (Gibco, Grand Island, NY, USA), supplemented with 10% foetal bovine serum (Gibco) and 1% (v/v) penicillin/streptomycin (Beyotime, Shanghai, China).

### 2.3 Establishment of stably transfected cell lines

The lentiviruses were obtained from Sangon (Shanghai, China). Stably transfected cells were established according to our previous studies and the manufacturer's instructions.^22^ Briefly, recombinant wild‐type BATF2 overexpression plasmids were constructed, and then mutated in the NES region of BATF2. Subsequently, HCT116 cells were infected with lentivirus‐packaged recombinant plasmids, and the resistant cells were selected with corresponding antibiotics to obtain stable cell lines. The cell lines with BATF2 overexpression were termed as C116 cells, while the stable cell lines with mutated BATF2 in the NES region were named A116 cells.

### 2.4 Animal study

Four to five‐week‐old female nude mice were purchased from Beijing Huafukang Bioscience (Beijing, China). To construct the xenograft mouse models, 5 × 10^6^ HCT116, C116 and A116 cells were subcutaneously injected into the dorsal area of the right flank of the mice separately. Tumour sizes were monitored every 3 days. After 27 days, mice were sacrificed for subsequent experiments. The in vivo study received approval from the Animal Welfare and Ethics Committee of Army Medical University (No. AMUWEC20173938).

### 2.5 Western blot analysis

Western blot analysis was performed as previously described.^12^, ^23^, ^24^ Total protein was extracted by using a cell lysis buffer (Beyotime), while nuclear protein was extracted with a nuclear and cytoplasmic protein extraction kit (Thermo Scientific, Rockford, IL, USA). Primary antibody against BATF2 was purchased from Abcam (Cambridge, MA, USA). Primary antibodies against CRM1, β‐actin and LaminB were purchased from Santa Cruz (Dallas, TX, USA). Primary antibodies against ubiquitin, pRb (Ser807/811), pRb (Ser795), CDK4, p21, cyclin A2, cyclin B1, cyclin D1 and cyclin E1 were purchased from Cell Signaling Technology (Boston, MA, USA).

### 2.6 Immunohistochemistry staining

Immunohistochemistry (IHC) staining was carried out using tumour samples from nude mice or patients as previously described.^22^, ^25^ The samples were incubated with primary antibodies against BATF2 (Abcam), CRM1 or Ki67 (Santa Cruz), followed by the corresponding secondary antibodies (Zhongshan Biotechnology, Beijing, China). The staining images were observed using Olympus IX81 photomicroscope. Scoring of IHC staining was performed in accordance with the immunoreactive score, calculated by using percentage of positive cells multiplied by staining intensity.^22^ Percentage of positive cells—negative: 0; 10%: 1; 11%−50%: 2; 51%−80%: 3; and >80%: 4. Staining intensity—negative: 0; weak: 1; moderate: 2; and strong: 3. The IHC signals were evaluated independently by three pathologists who were blinded to the patients' information.

### 2.7 Co‐immunoprecipitation

Co‐immunoprecipitation (co‐IP) was performed by using an immunoprecipitation kit (Novex, Life Technologies, Carlsbad, CA USA). Briefly, immunoglobulin G (IgG) or BATF2 antibody (2 µg) was incubated with Dynabeads protein G with rotation for 1 h and immunoprecipitated with 500 µg protein at 4°C overnight. After elution with elution buffer, the supernatant was collected for Western blot or proteomic analysis. IgG served as negative control, while total protein served as the input.

### 2.8 Label‐free liquid chromatography–mass spectrometry/mass spectrometry proteomic analysis

Proteomic analysis was performed as previously described.^26^, ^27^ Briefly, the co‐immunoprecipitated proteins of CCD 841 CoN and HCT116 cells eluted from anti‐IgG or anti‐BATF2 magnetic beads were subjected to liquid chromatography–mass spectrometry/mass spectrometry (LC–MS/MS) assays by Genecreate Technology (Wuhan, China). The proteomics data were analysed by using ProteinPilot Software v4.5, with Paragon algorithm integrated for protein search against human UniProt database. The protein sequences were mapped with Gene Ontology terms by using blast2go v4.5 pipeline5, with an *e*‐value less than 1 × 10^−5^. The proteomics data have been submitted to the ProteomeXchange database (http://proteomecentral.proteomexchange.org) via iProX repository (No. PXD037788).

### 2.9 Laser scanning confocal microscopy

The paraformaldehyde‐fixed cells were incubated with BATF2 antibody overnight and subsequently Cy3‐conjugated goat anti‐rabbit antibody for 1 h in the dark. After incubation with DAPI for 1 min, the signals were imaged using a confocal fluorescence microscope (Zesis, Oberkochen, Germany).

### 2.10 Bioinformatic analysis of the interaction between BATF2 and CRM1

Bioinformatic prediction of the binding between BATF2 and CRM1 was analysed by using ZDOCK software. The structure of CRM1 is the α chain of PDB files with PDB number 5dis, while BATF2 is a structure file obtained by homologous modelling with PDB number 2qpw as the model. The complex model was selected according to the docking results of ZDOCK software by using electrostatics, shape complementarity and IFACE statistical potential.^28^ Furthermore, the interaction network diagram was performed by using ligplot software.^29^

### 2.11 Gene knockdown by siRNA

siRNAs against CRM1, BATF2 and scramble siRNAs were purchased from Santa Cruz and transfected into HCT116 cells by using Lipofectamine 2000 (Invitrogen, Carlsbad, CA, USA).

### 2.12 Cell viability assay

Cells were plated onto 96‐well plates, and cell viability was determined daily for the indicated days by using a Cell Counting Kit‐8 (CCK‐8) kit (Dojindo Lab., Kumamoto, Japan).

### 2.13 Clonogenic capacity

Cells were seeded onto six‐well plates and then cultured for 10 days for staining using the crystal violet. The clone number was counted by using ImageJ software.

### 2.14 Cell cycle detection

The ethanol‐fixed cells were incubated with propidium iodide (Invitrogen) for 30 min and then harvested for flow cytometry analysis (BD Biosciences, San Jose, CA, USA).

### 2.15 Luciferase reporter gene assay

pAP‐1‐Luc (BD Biosciences, Oxford, UK) was co‐transfected with β‐gal vector (Promega, Madison, WI, USA) into HCT116, C116 and A116 cells using Lipofectamine 2000. Cells were analysed by using a luciferase assay kit (Promega).^12^ β‐Gal was determined by using a β‐gal assay kit (Beyotime) and served as an internal control.

### 2.16 Electrophoretic mobility shift assay

Electrophoretic mobility shift assay (EMSA) was performed by using an EMSA kit (Pierce, Rockford, IL, USA) as we previously described.^12^ The biotin‐labelled AP‐1 probe was first incubated with negative control or nuclear proteins of HCT116, C116 or A116 cells separately for 20 min at 25°C. The mixtures were electrophoresed and transferred to a nylon membrane for crosslinking and blocking. Then, the nylon membrane was incubated with horseradish peroxidase‐conjugated streptavidin and assayed with chemiluminescent substrate. AP‐1 probe: 5′‐CGCTTGATGACTCAGCCGGAA‐3′. The consensus binding sites for AP‐1 are underlined, through which AP‐1 directly binds to the promoter region of its downstream genes.

### 2.17 Statistical analysis

Data are expressed as the mean ± SD. Comparisons were analysed by using a two‐tailed unpaired *t* test or one‐way analysis of variance (ANOVA). Tumour volumes were tested by using two‐way ANOVA. Overall survival (OS) was analysed by using a Kaplan–Meier method. Correlation between clinical parameters and BATF2 or CRM1 expression was determined by using Pearson's chi‐squared test. The scatter diagram was drawn by using the ggplot2 program package of R language (version 4.2.2). Statistical analyses were performed using GraphPad Prism (version 8.0) software. *p* < .05 was considered statistically significant.

## 3 RESULTS

### 3.1 Cytoplasmic BATF2 expression negatively correlates with the prognosis of CRC patients

**FIGURE 1:**
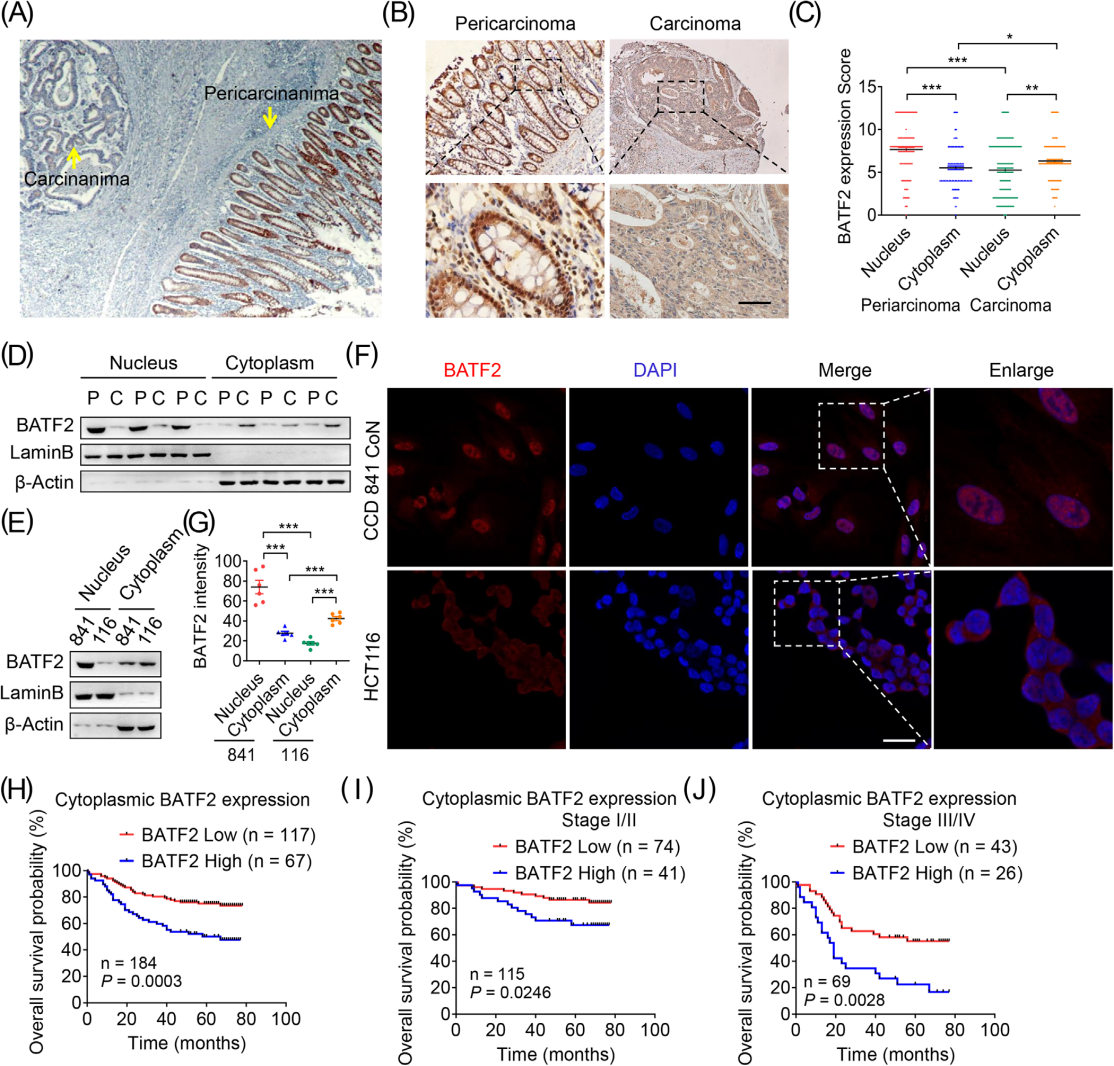
Cytoplasmic BATF2 expression negatively correlates with the prognosis of colorectal cancer (CRC) patients. (A–C) Representative immunohistochemistry (IHC) staining of BATF2 expression in CRC and pericarcinoma tissues from 184 CRC patients (A and B), and quantification of IHC images by using ImageJ software (C). Scale bar: 100 µm. (D and E) Western blot analysis of nuclear and cytoplasmic BATF2 expression in fresh frozen CRC and pericarcinoma tissues from three CRC patients (D) and CCD 841 CoN and HCT116 cells (E). C: carcinoma; P: pericarcinoma. (F and G) Immunofluorescence analysis of BATF2 in CCD 841 CoN and HCT116 cells (F) and quantification of BATF2 expression by using ImageJ software (G). Scale bar: 10 µm. (H–J) Kaplan–Meier estimates of overall survival time based on cytoplasmic BATF2 expression levels from 184 CRC patients, both at stage I/II (*n* = 115) and stage III/IV (*n* = 69). Data are expressed as mean ± SD. ^*^ *p <* .05, ^**^ *p* < .01, ^***^ *p* < .001.

The cytoplasmic localisation of BATF2 was observed in multiple tumours, including CRC, liver cancer, bile duct cancer, gastric cancer, kidney cancer, lung adenocarcinoma, lung adenosquamous carcinoma, lung squamous cell carcinoma, nasopharyngeal carcinoma and pancreatic adenocarcinoma (Figures 1A,B and S1). Furthermore, CRC and pericarcinoma tissues from 184 patients were collected to investigate the subcellular distribution of BATF2. Statistical analysis revealed that the cytoplasmic BATF2 expression levels were higher in CRC than those in pericarcinoma tissues, which was contrary to its nuclear expression levels (Figure 1C). To further validate these observations, the nuclear and cytoplasmic proteins from three cases of fresh frozen CRC and pericarcinoma tissues were isolated for Western blot analysis, exhibiting higher BATF2 expression in the cytoplasm of tumour tissues than that in pericarcinoma (Figure 1D), which was also observed in human CRC cells (HCT116) compared with normal colon epithelial cells (CCD 841 CoN) (Figure 1E–G).

To investigate the biological role of various distribution of BATF2, comparisons were analysed between BATF2 expression levels and clinical parameters of 184 CRC patients. Although BATF2 expression had no significant correlation with the gender, age, diagnosis or classification (Table S1), higher cytoplasmic BATF2 expression level was associated with poorer OS probability in CRC patients (*p* = .0003), both at stage I/II (*p* = .0246) and stage III/IV (*p* = .0028) (Figure 1H–J), while higher total or nuclear BATF2 expression was correlated with longer OS in CRC patients (*p* < .05) (Figure S2A–D). These data indicated a negative correlation between cytoplasmic BATF2 expression and CRC prognosis.

### 3.2 BATF2 is degraded by ubiquitination after translocating to cytoplasm in CRC

**FIGURE 2:**
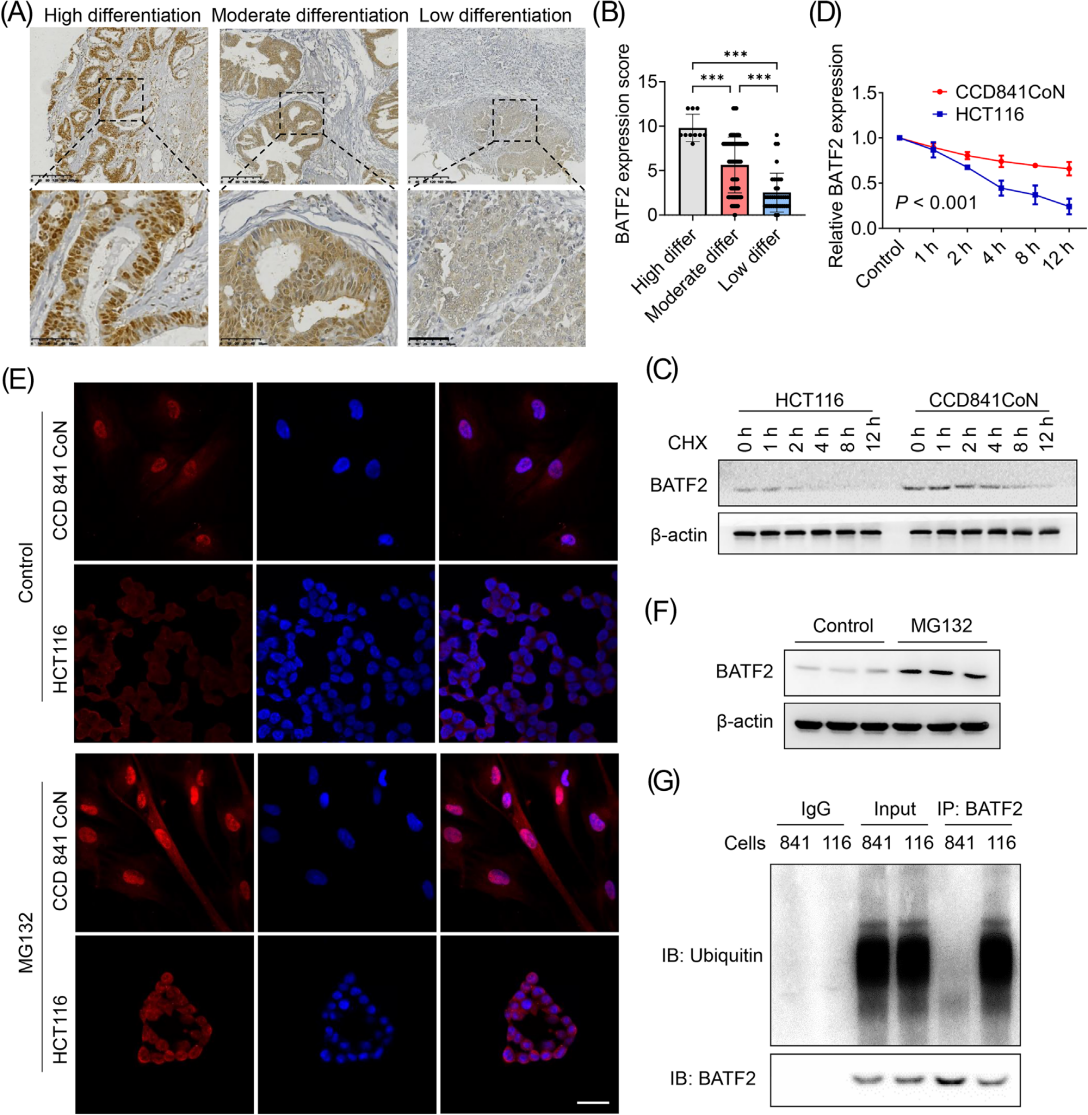
BATF2 is degraded by ubiquitination after translocation to cytoplasm in colorectal cancer (CRC) cells. (A and B) Representative immunohistochemistry (IHC) staining of BATF2 in CRC tissues with high, moderate and low differentiation (A) and the quantification analysis using ImageJ software (B). (C and D) Western blot analysis of BATF2 expression in CCD 841 CoN and HCT116 cells incubated with 10 µM cycloheximide (CHX), a translational inhibitor, for the indicated time (C) and the quantification analysis using ImageJ software (D). (E) Immunofluorescence assay and Western blot analysis of BATF2 expression in CCD 841 CoN and HCT116 cells treated with control or 10 µM MG132, a proteasome inhibitor. Scale bar: 10 µm. (F) Western blot analysis of BATF2 expression in HCT116 cells treated with control or MG132. (G) Co‐immunoprecipitation (co‐IP) analysis of the binding between BATF2 and ubiquitin in CCD 841 CoN and HCT116 cells, taking immunoglobulin G (IgG) as a negative control and total protein as a positive control. 841: CCD 841 CoN; 116: HCT116. Data are expressed as mean ± SD. ^***^ *p* < .001.

Further analysis showed that nuclear BATF2 expression decreased with the increasing degree of malignancy in human CRC samples (Figure 2A,B). BATF2 expression was higher in human CRC samples with high differentiation, and much lower in those with low differentiation (Figure 2A,B). Therefore, we hypothesised that BATF2 might gradually degrade along with CRC progression. To validate this hypothesis, BATF2 protein turnover rate in HCT116 and CCD 841 CoN cells was examined using a translational inhibitor, cycloheximide, which could inhibit the de novo protein synthesis. It was found that the half‐life of BATF2 protein in CCD 841 CoN cells was much longer than that in HCT116 cells (*p* < .001) (Figure 2C,D). Then, we treated CCD 841 CoN and HCT116 cells with a classic proteasome inhibitor, MG132, since ubiquitin–proteasome pathway leads to the degradation of the majority of cellular proteins.^30^ Immunofluorescence assay and Western blot analysis showed that BATF2 expression significantly increased upon MG132 treatment in HCT116 cells (Figure 2E,F). To further confirm our hypothesis, co‐IP analysis was carried out, which demonstrated much more ubiquitinated BATF2 protein in HCT116 cells than that in CCD 841 CoN cells (Figure 2G). These results collectively suggested that BATF2 was degraded by ubiquitination after translocating to cytoplasm in CRC.

### 3.3 Cytoplasmic localisation of BATF2 is mediated by CRM1‐dependent nuclear export

**FIGURE 3:**
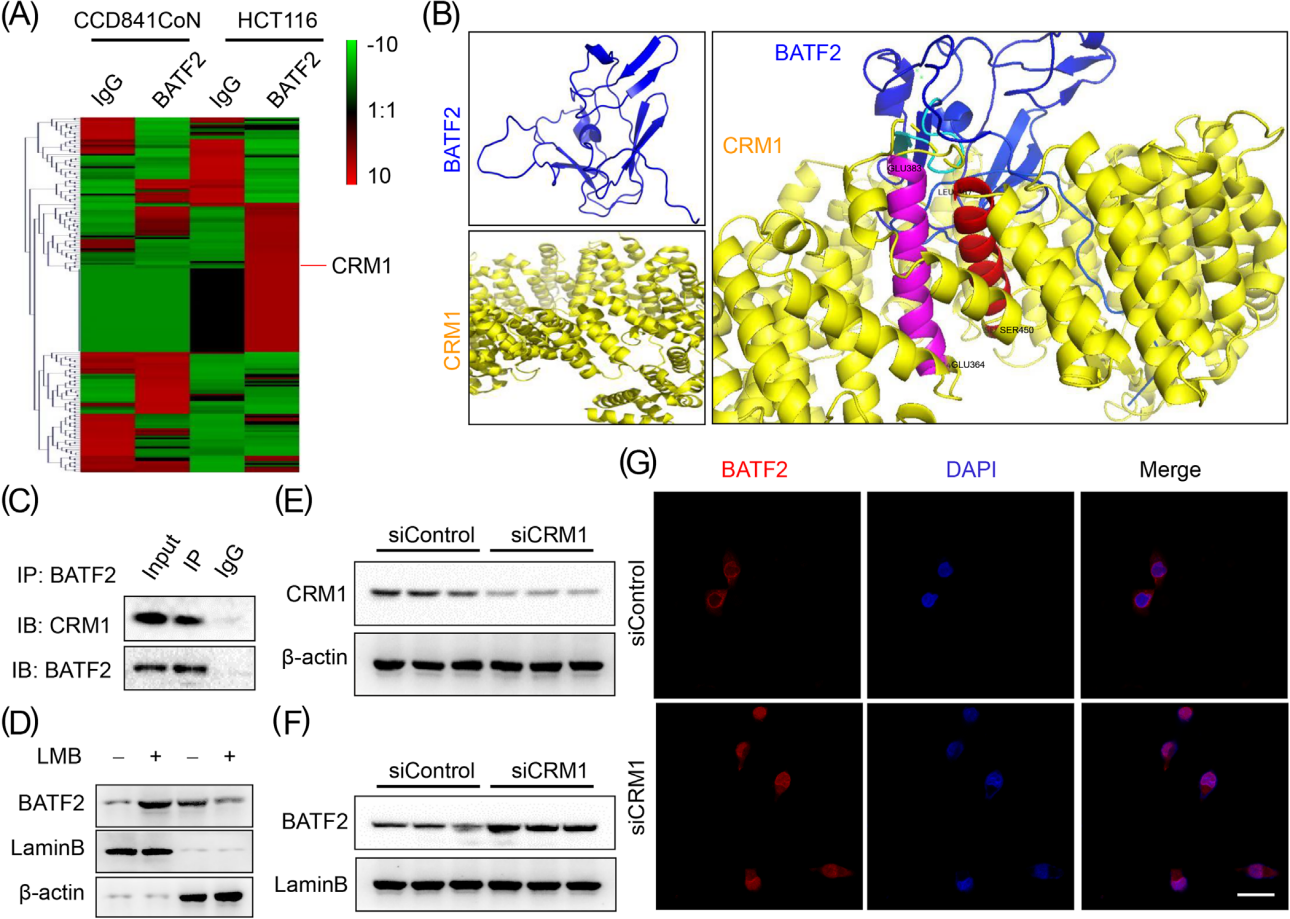
Cytoplasmic localisation of BATF2 is mediated by chromosome region maintenance 1 (CRM1)‐dependent nuclear export. (A) The heatmap of label‐free liquid chromatography–mass spectrometry/mass spectrometry (LC–MS/MS) proteomic analysis of the co‐immunoprecipitated proteins in CCD 841 CoN and HCT116 cells by using immunoglobulin G (IgG) and BATF2 antibodies separately. (B) Bioinformatic prediction of the binding between BATF2 and CRM1 by using a ZDOCK method. Important amino acids of CRM1 are highlighted in pink, including GLU383 to GLU364, and red, including LEU467 to SER450. Other structures of CRM1 are highlighted in yellow, and structures of BATF2 are highlighted in blue. (C) Co‐immunoprecipitation (co‐IP) analysis of the binding between BATF2 and CRM1 in HCT116 cells. (D) Western blot analysis of the nuclear and cytoplasmic BATF2 expression in HCT116 cells incubated with control or a CRM1 inhibitor, leptomycin B (LMB). (E–G) Western blot and immunofluorescence analysis of the nuclear BATF2 expression in HCT116 cells transfected with control siRNAs (siControl) or siRNAs against CRM1 (siCRM1). Scale bar: 10 µm.

To screen the candidate proteins that mediate the translocation of BATF2, we performed co‐IP and label‐free LC–MS/MS proteomic analysis, which showed that CRM1 protein was substantially higher in BATF2‐immunoprecipitated proteins than that in IgG group, and the BATF2‐bound CRM1 protein was significantly higher in HCT116 cells than that in CCD 841 CoN cells (Figure 3A). As reported, NCT is commonly accomplished with the help of nuclear transport proteins,^31^ while CRM1 is a major nuclear export protein that is responsible for transporting cargo proteins out of nucleus.^8^, ^9^, ^10^ Therefore, we explored the interactions between BATF2 and CRM1. Bioinformatic analysis predicted a direct binding between BATF2 and CRM1 by using ZDOCK software (Figure 3B). Meanwhile, the interaction network diagram was performed by using ligplot software, revealing the hydrogen bond through which BATF2 may interact with CRM1 (Figure S3). To verify these predictions, we performed co‐IP assay, which suggested that BATF2 could directly bind to CRM1 (Figure 3C). To confirm whether the transport of BATF2 was mediated by CRM1, we treated HCT116 cells with a CRM1 inhibitor, leptomycin B, which significantly enhanced the nuclear accumulation of BATF2 in HCT116 cells (Figure 3D). Subsequent Western blot and immunofluorescence analysis also showed an increased nuclear BATF2 expression in HCT116 cells after the knockdown of CRM1 expression using siRNAs (Figure 3E–G). These data revealed that cytoplasmic localisation of BATF2 was mediated by CRM1‐dependent nuclear export.

### 3.4 CRM1 negatively correlates with nuclear expression of BATF2 and CRC prognosis

**FIGURE 4:**
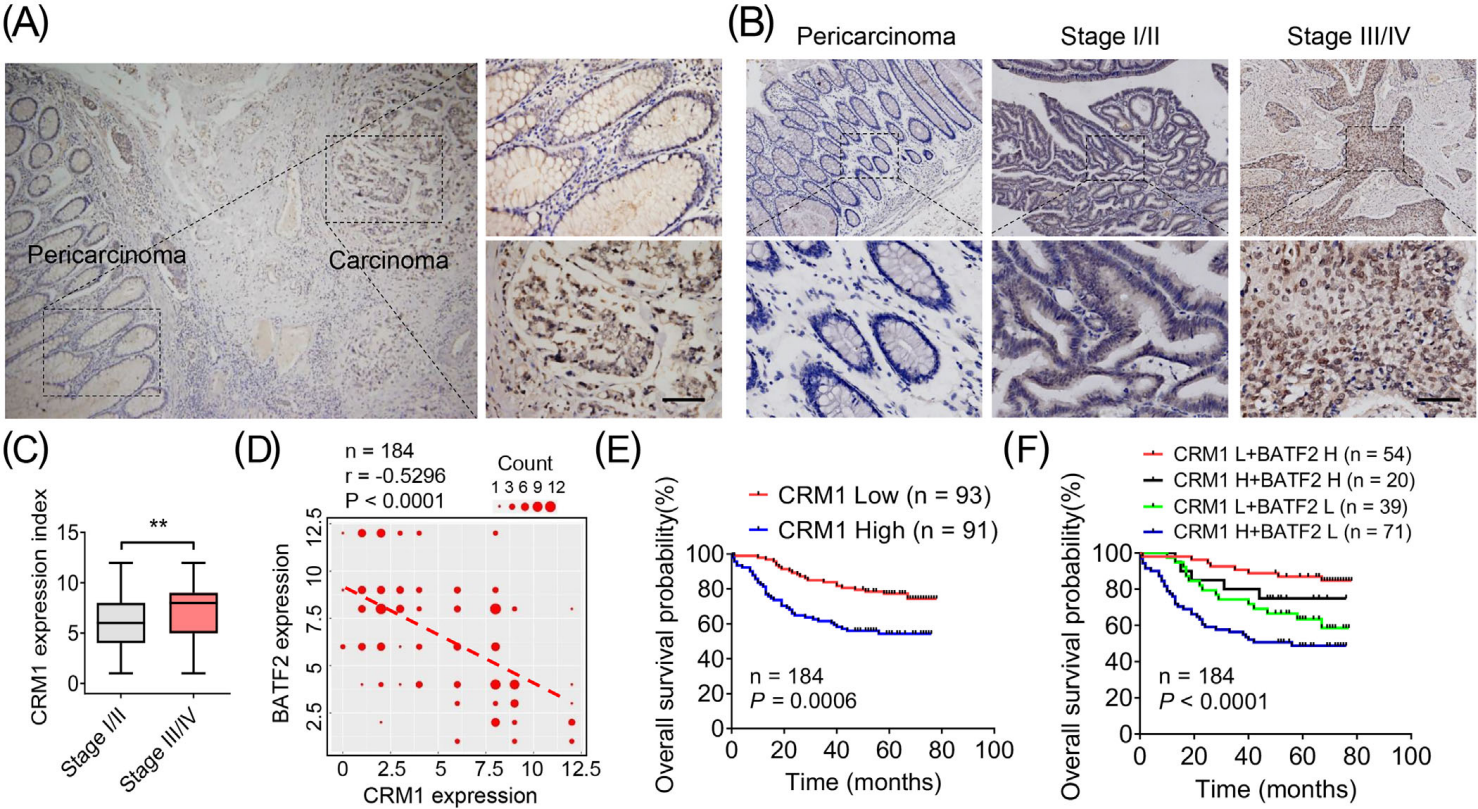
Chromosome region maintenance 1 (CRM1) negatively correlates with nuclear expression of BATF2 and colorectal cancer (CRC) prognosis. (A–C) Representative immunohistochemistry (IHC) staining of CRM1 in normal and CRC samples with different differentiation (A and B) and the quantification analysis between early stage (I/II) and advanced stage (III/IV) CRC tissues using ImageJ software (C). Scale bar: 100 µm. (D) The negative correlation between the protein levels of BATF2 and CRM1. The scatter diagram was drawn by using the ggplot2 program package of R language (version 4.2.2). The number of repeated dots was represented by Count. (E) Kaplan–Meier analysis of survival time based on the expression level of CRM1 in 184 CRC patients. (F) Kaplan–Meier estimates of survival time based on the expression levels of CRM1 and BATF2 in 184 CRC patients. H: high; L: low. ^**^ *p* < .01.

To further investigate the association between CRM1 and BATF2, IHC was used to assess their expression levels in CRC samples. It was found that CRM1 expression was much higher in CRC tissues than that in adjacent colon tissues (Figure 4A). CRM1 expression was also higher in CRC tissues at advanced stages (III/IV) compared with early stages (I/II) (*p* = .0026) (Figure 4B,C). Of note, statistical analysis revealed a moderately negative correlation between the expressions of CRM1 and nuclear BATF2 (*r* = −0.5296, *p* < .0001) (Figure 4D), implying a potential CRM1–BATF2 signalling pathway in CRC. Furthermore, patients with higher CRM1 expression levels had shorter survival time than those with lower CRM1 expression levels (*p* = .0006) (Figure 4E). To determine whether CRM1 in combination with BATF2 can serve as a more accurate prognostic marker, 184 human CRC samples were assigned into four groups based on the expression levels of CRM1 and BATF2 (Figure 4F). The OS time was the longest in the low CRM1 but high BATF2 group and the shortest in high CRM1 but low BATF2 group compared with the other two groups (*p* < .0001) (Figure 4F).

### 3.5 NES mediates the nuclear export of BATF2

**FIGURE 5:**
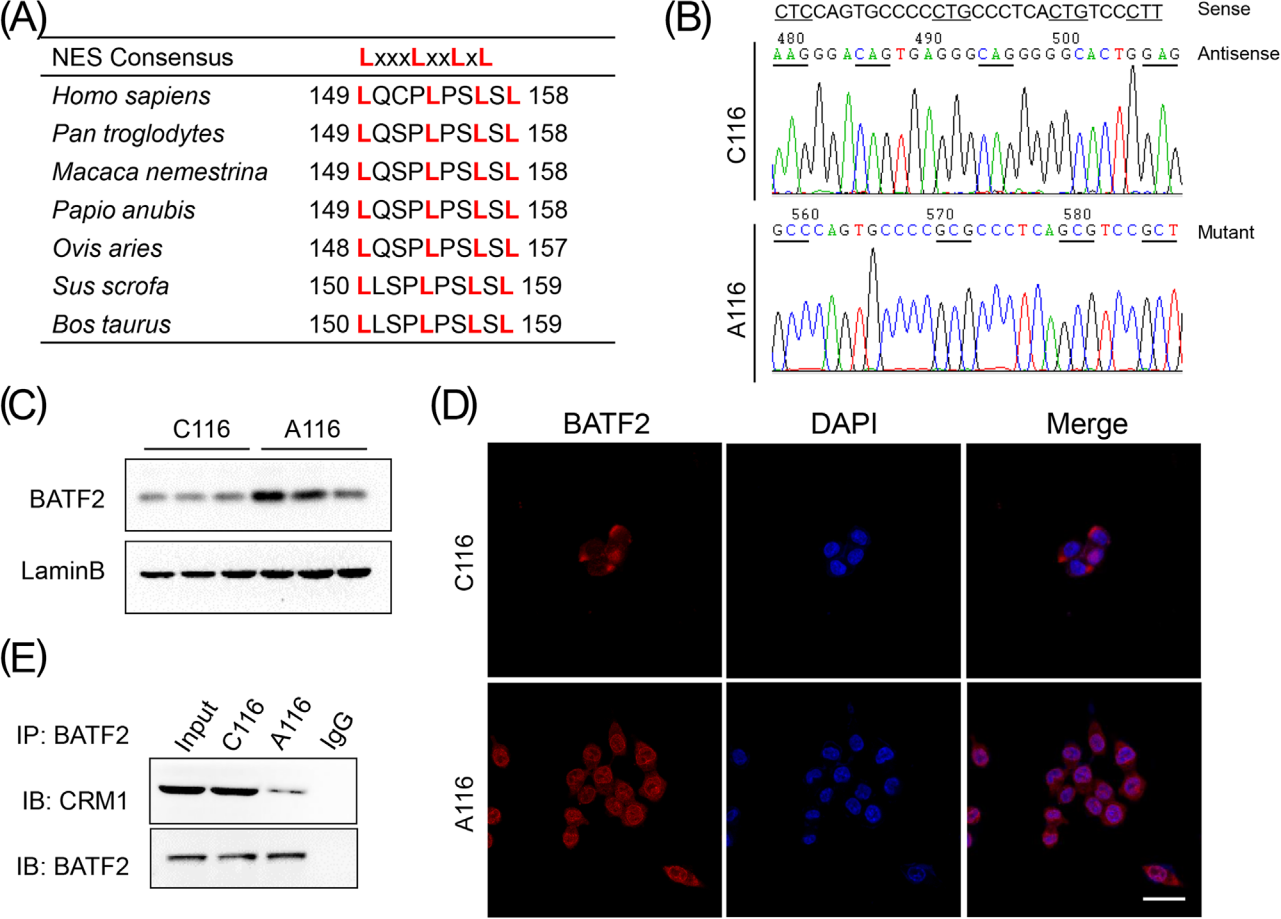
Chromosome region maintenance 1 (CRM1) negatively correlates with nuclear expression of BATF2 and colorectal cancer (CRC) prognosis. (A) Bioinformatic analysis of the conserved nuclear export sequence (NES) region (any amino acid) in *BATF2* protein in mammals by using BLAST software of national center of biotechnology information (NCBI). (B) Recombinant BATF2 plasmids without mutation (C116) or with leucine mutated into alanine in the NES region (A116) were separately infected into HCT116 cells by using lentivirus. (C and D) Western blot and immunofluorescence analysis of nuclear BATF2 expression in C116 and A116 cells. (E) Co‐immunoprecipitation (co‐IP) analysis of the binding between CRM1 and BATF2 in C116 and A116 cells. Scale bar: 10 µm.

To illuminate the mechanism of nuclear export of BATF2, bioinformatic analysis was performed, which identified a classical NES region in the amino acids of *BATF2* protein that was highly conserved in several mammals, suggesting similar biological functions among mammals (Figure 5A). Recombinant BATF2 plasmids without mutation (C116) or with leucine mutated into alanine in the NES region (A116) were separately infected into HCT116 cells by using lentivirus (Figure 5B). After the mutation of NES, nuclear BATF2 expression was much higher in A116 cells than that in C116 cells (Figure 5C). As expected, BATF2 localised mainly in the nucleus of A116 cells, rather than in C116 cells (Figure 5D). Moreover, the interaction between BATF2 and CRM1 was significantly attenuated in A116 cells compared with C116 cells (Figure 5E). These results demonstrated that the NES region in BATF2 was essential for CRM1‐mediated translocation of BATF2.

To further explore the nuclear localisation signal (NLS) of BATF2, the Identification of Nucleus Signal Peptide software^32^ was used, which predicted an NLS (RGKLG) region (Figure S4A). When the NLS was mutated into PADQP, subsequent immunofluorescence assay showed that BATF2 mainly accumulated in the cytoplasm, rather than the nucleus, suggesting that part of the entry of BATF2 into the nucleus was blocked by the mutation in its NLS region (Figure S4A,B).

### 3.6 Suppression of BATF2 nuclear export represses CRC growth in vitro and in vivo

**FIGURE 6:**
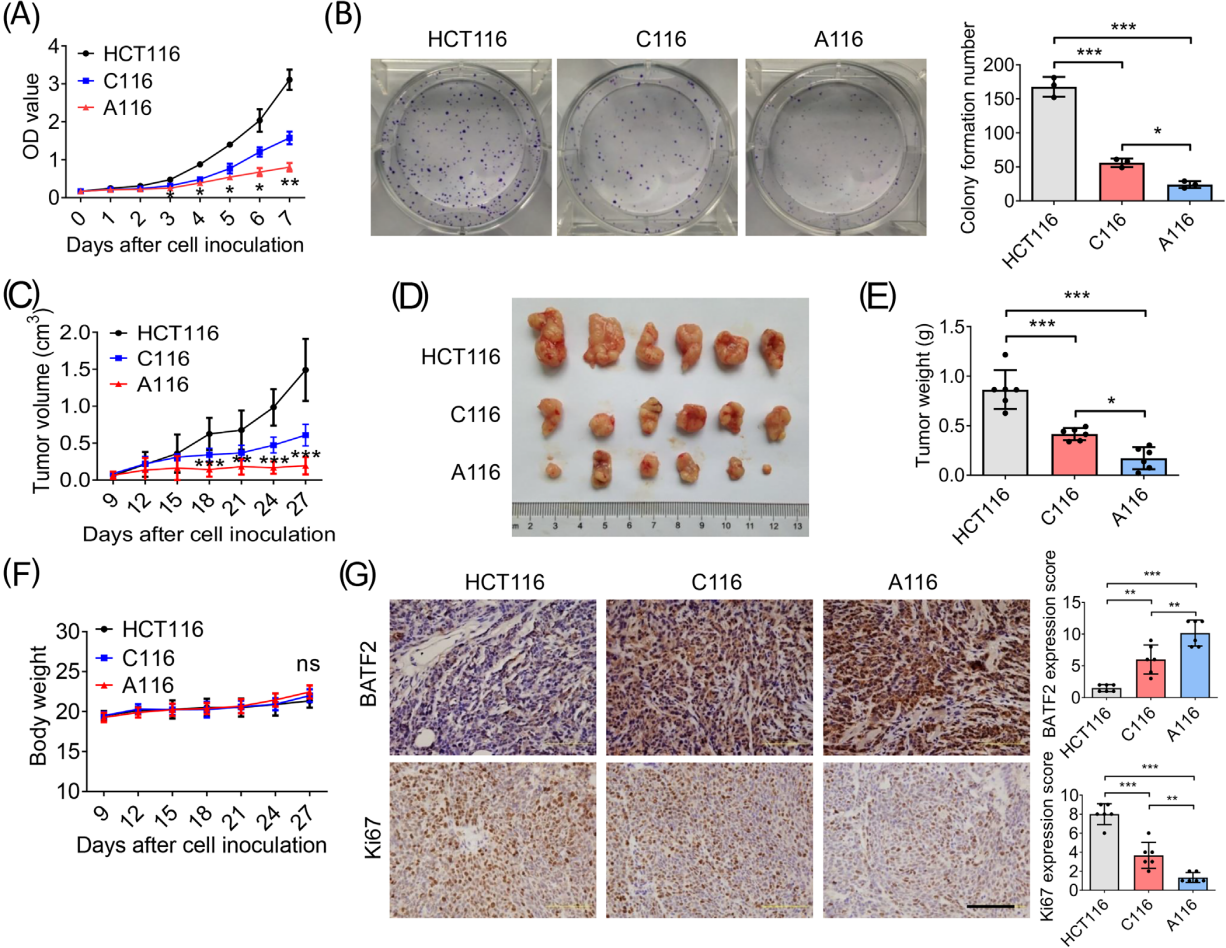
Suppression of BATF2 nuclear export represses colorectal cancer (CRC) growth in vitro and in vivo. (A) The cell proliferation of HCT116, C116 and A116 cells at the indicated times was determined by using CCK‐8 assay. (B) The colony forming ability of HCT116, C116 and A116 cells was measured by using colony formation assay. (C–G) Xenograft nude mouse models were constructed by subcutaneously injecting 5 × 10^6^ HCT116, C116 or A116 cells separately (*n* = 6 per group). (C) The tumour volume was calculated every 3 days starting on the 9th day, and the mice were sacrificed 27 days after cell inoculation. (D and E) The xenografts were excised for the comparisons of tumour size and weight. (F) The body weights of the mice were recorded. (G) Immunohistochemistry (IHC) analysis of the expression levels of BATF2 and Ki67 in HCT116, C116 and A116 groups, and the quantification of IHC images was analysed by using ANOVA. *n* = 6 per group. Data are expressed as mean ± SD. ns: no significance. ^*^ *p <* .05, ^**^ *p* < .01, ^***^ *p* < .001.

Subsequently, the proliferation of HCT116, C116 and A116 cells was determined by using CCK‐8, showing lower proliferation in C116 cells and the lowest proliferation in A116 cells (Figure 6A), which was also confirmed by colony formation assay (Figure 6B). To investigate the role of BATF2 in vivo, xenograft nude mouse models were constructed by separately injecting with HCT116, C116 or A116 cells. Compared with the HCT116‐injected mice, both tumour volume and weight were smaller in C116 group and the smallest in A116 group in the xenograft of nude mice (Figure 6C–E), while their body weight had no significant change (Figure 6F). As anticipated, BATF2 expression was higher in C116 group and the highest in A116 group, accompanied with a low expression level of Ki67, a biomarker of cell proliferation (Figure 6G). These data revealed that suppression of BATF2 nuclear export represses CRC growth.

### 3.7 Inhibition of BATF2 nuclear export induces G1 phase arrest through AP‐1/cyclin D1/pRb signalling pathway

**FIGURE 7:**
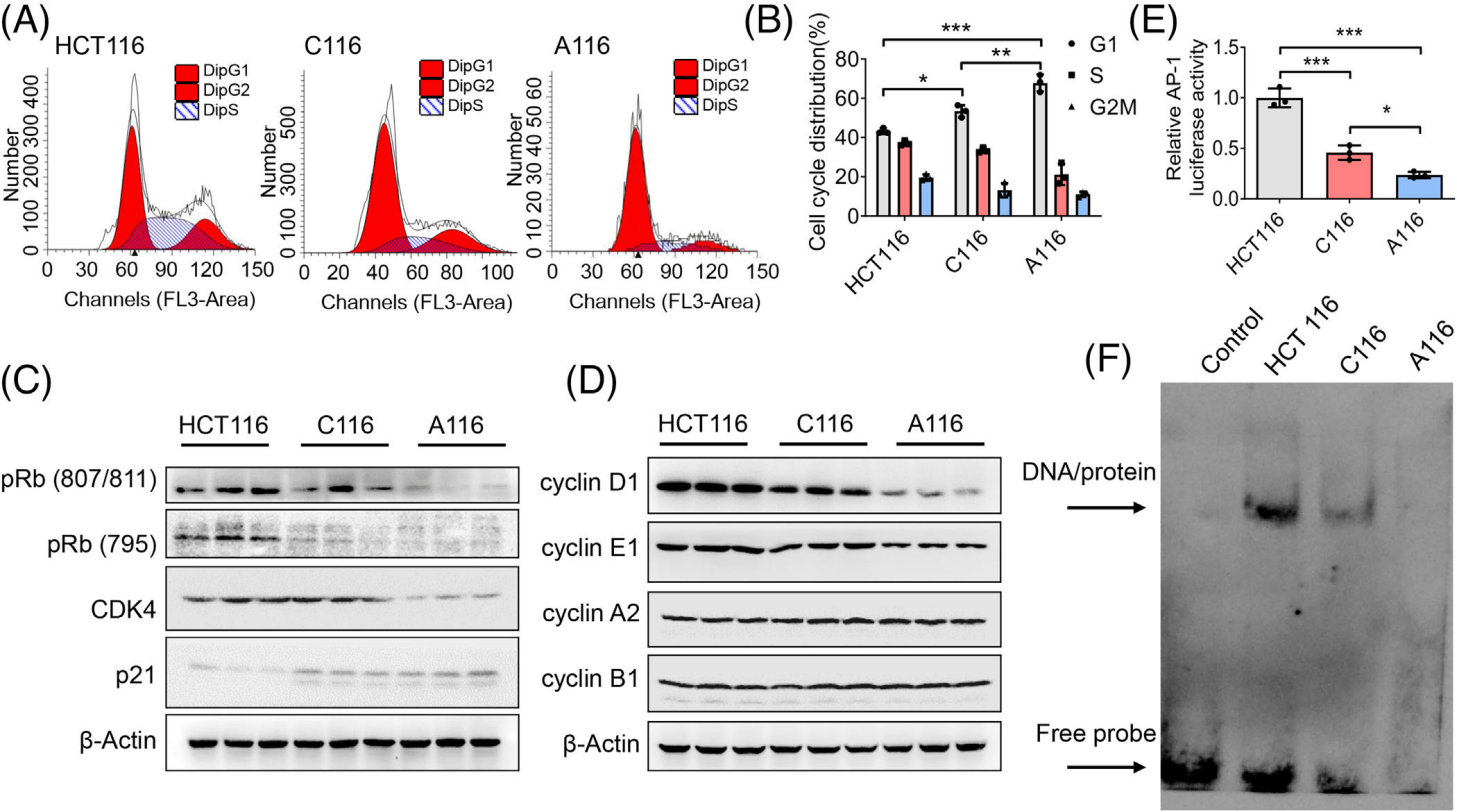
Inhibition of BATF2 nuclear export induces cell cycle arrest through suppressing activator protein 1 (AP‐1)/cyclin D1/ phosphorylated retinoblastoma protein (pRb) signalling pathway. (A and B) Cell cycle of HCT116, C116 and A116 cells was determined by using flow cytometry. (C) Western blot analysis of cell cycle checkpoint‐related genes, including pRb (Ser807/811), pRb (Ser795), CDK4 and p21. (D) Western blot analysis of cell cycle markers, including cyclin A2, cyclin B1, cyclin D1 and cyclin E1. (E) pAP‐1‐Luc was co‐transfected with β‐gal vector into HCT116, C116 and A116 cells using Lipofectamine 2000 for luciferase reporter gene assay, which was normalised against β‐gal activity. (F) Electrophoretic mobility shift assay (EMSA) analysis of the DNA binding activity of AP‐1 in HCT116, C116 and A116 cells. Data are expressed as mean ± SD. ^*^ *p <* .05, ^**^ *p* < .01, ^***^ *p* < .001.

Cell cycle of HCT116, C116 and A116 cells was determined by using flow cytometry, revealing G1 phase arrest in C116 cells, which was more serious in A116 cells (Figure 7A,B). Therefore, canonical cell cycle checkpoint‐related genes^33^ were analysed by using Western blotting, which demonstrated that the expressions of G1/S transition‐regulating proteins, including pRb and cyclin‐dependent kinase 4 (CDK4), were inhibited, while p21 was induced in C116 cells, with a further acceleration in A116 cells (Figure 7C). The expressions of cyclin D1 and cyclin E, the marker of G1 phase of the cell cycle, had the same tendency in C116 and A116 cells (Figure 7D). Since cyclin D1 was a classical target gene of AP‐1,^34^ we transfected pAP‐1‐Luc reporter plasmids into HCT116, C116 and A116 cells respectively. Cells were collected for luciferase reporter gene assay, which showed a reduced AP‐1 activity in C116 cells, with a further reduction in A116 cells (Figure 7E). Then, nuclear proteins of HCT116, C116 and A116 cells were extracted for EMSA. As shown in Figure 7F, the biotin‐labelled AP‐1 probe yielded a DNA/protein band with nuclear proteins of HCT116 cells, which was suppressed in C116 cells, with a further suppression in A116 cells. These findings indicated that AP‐1 transcriptional activity is inhibited by the inhibition of BATF2 nuclear export.

To further demonstrate the tumour suppressive effect of BATF2 in CRC, we performed knockdown tests by using siRNAs against BATF2 (siBATF2). CCK‐8 assay showed that the proliferation of HCT116 cells transfected with siBATF2 was significantly faster than those transfected with scramble siRNAs (siControl) (Figure S5A,B). Besides, luciferase reporter gene assay and EMSA also exhibited an enhanced AP‐1 activity in HCT116 cells transfected with siBATF2 compared with those transfected with siControl (Figure S5C,D). Notably, to investigate the correlations between BATF2 and AP‐1 in primary samples, we carried out IHC staining using human CRC samples, which revealed that the expression of c‐Jun, the AP‐1 major subunit, was low in highly differentiated tissues, but high in poorly differentiated tissues (Figure S6A,B). Further statistical analysis revealed that c‐Jun was negatively correlated with the expression level of nuclear BATF2 (*r* = −0.5273, *p* < .0001) (Figure S6C). These findings collectively suggested that inhibiting the translocation of BATF2 induces G1 phase arrest through suppressing AP‐1/cyclin D1/pRb signalling pathway.

## 4 DISCUSSION

**FIGURE 8:**
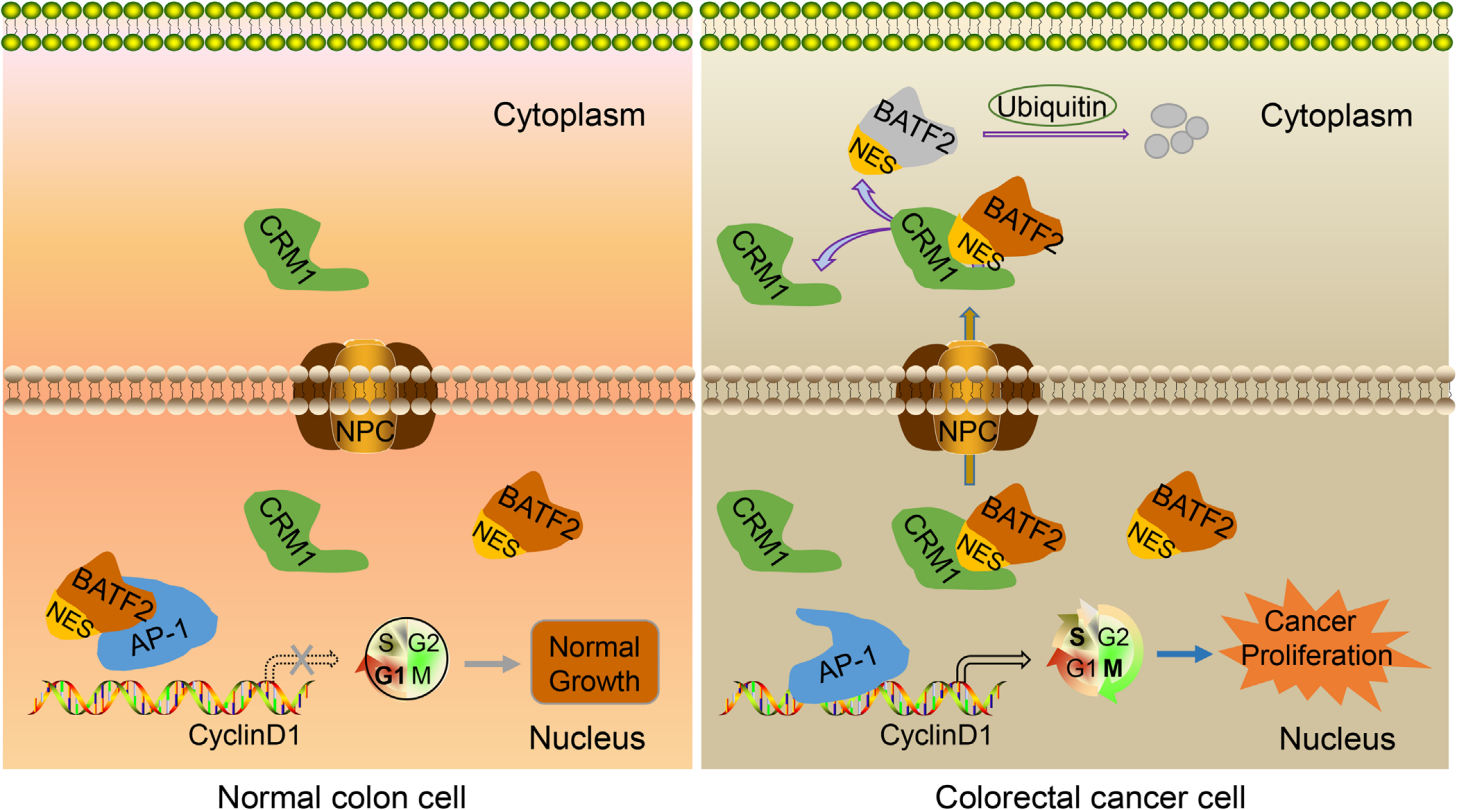
A proposed model elucidating the mechanism by which nuclear export of BATF2 promotes colorectal cancer (CRC) progression. In normal colon cells, BATF2 suppresses activator protein 1 (AP‐1) activity and its downstream oncogene expression, keeping cells from over‐proliferation. In CRC cells, the highly expressed chromosome region maintenance 1 (CRM1) interacts with BATF2 to form a complex via directly binding to its nuclear export sequence (NES) region, which translocates into the cytoplasm, leading to an ubiquitin‐mediated degradation of BATF2 and subsequent activated AP‐1‐driven cell proliferation, ultimately contributing to cancer growth.

Emerging studies, including ours, have suggested that BATF2 is a tumour suppressor, suppressing the growth and metastasis of multiple tumours mainly through binding to AP‐1, thereby regulating the expressions of the downstream genes.^11^, ^12^, ^13^, ^14^ Previous reports have shown that BATF2 was located in the nucleus.^11^, ^12^, ^13^, ^14^, ^15^, ^16^, ^17^, ^18^, ^19^, ^20^ However, the phenomenon, mechanism and biological functions of different subcellular localisations of BATF2 have not yet been reported. The present study for the first time demonstrated the abnormal nucleus–cytoplasmic distribution of BATF2 in CRC and proved the negative correlation between cytoplasmic BATF2 expression and prognosis of CRC patients. Furthermore, we identified the NES region of BATF2 and elucidated the mechanism by which nuclear export of BATF2 was mediated by its interaction with CRM1 through its NES region, leading to CRC proliferation in vitro and in vivo (Figure 8).

Of note, we found that cytoplasmic localisation of BATF2 existed in not only CRC samples but also other tumour tissues, including liver cancer, bile duct cancer, gastric cancer, kidney cancer, lung adenocarcinoma, lung adenosquamous carcinoma, lung squamous cell carcinoma, nasopharyngeal carcinoma and pancreatic adenocarcinoma (Figure S1). Interestingly, previous studies have already observed cytoplasmic expression of BATF2 in CRC,^14^ hepatocellular carcinoma^16^ and lung cancer^35^ in their IHC assays, although these reports ignored the abnormal cytoplasmic distribution of BATF2 and the underlying mechanisms. Actually, the export of specific proteins from nucleus is essential for delicately balancing cell growth and death in both normal and malignant cells.^31^ Cancer cells frequently utilise this process to change the subcellular localisation of TSGs, such as p53 and p27, to promote cancer initiation and progression.^6^, ^7^, ^8^, ^9^, ^10^, ^31^ Surprisingly, TSGs have opposing roles during malignant transformation that are dependent on their various subcellular localisations, since nuclear export of TSGs ultimately contributes to tumour progression.^7^, ^36^, ^37^, ^38^ Therefore, elucidating the role and mechanism of nuclear export of TSGs may help explore novel therapeutic targets for cancer therapy.^31^

Mechanistically, after bioinformatic analysis, screening and experimental verification, we demonstrated that BATF2 was transported out of nucleus through interacting with CRM1, since siRNA‐mediated knockdown of CRM1 expression resulted in the nuclear accumulation of BATF2. As reported, nuclear export is commonly achieved with the help of transport receptors, which is a process of active transport.^8^, ^9^, ^10^ CRM1 serves as a major nuclear transporter that specifically recognises and binds to the NES region of cargo proteins to form a complex, thereby transporting them out of the nucleus.^8^, ^9^, ^10^ In this study, we identified a conserved NES region in *BATF2*, which provides fundamental conditions for its nuclear export. As anticipated, mutations of NES region in *BATF2*, converting leucine to alanine, significantly repressed its binding to CRM1 and suppressed the nuclear export of BATF2 and subsequent CRC proliferation in vitro and in vivo. These findings not only revealed the nuclear export of BATF2 and its biological effects but also elucidated the underlying mechanism and provided a novel therapeutic approach against CRC.

In the present study, we found that CRM1 expression was much higher in CRC tissues than that in pericarcinoma, which was also confirmed in other reports,^8^, ^9^ suggesting that CRM1 could serve as a potential therapeutic target for cancer therapy. Inspiringly, the selective inhibitors of CRM1, especially selinexor, have been recently developed as anticancer drugs.^8^, ^38^, ^39^ Based on data from several clinical trials, selinexor has been approved by the Food and Drug Administration for multiple myeloma (MM) and diffuse large B‐cell lymphoma,^8^, ^38^, ^40^, ^41^ and extensive researches on solid tumours have been conducted.^8^, ^42^ However, 89% of patients experienced serious drug‐related adverse events and side effects such as anaemia, thrombocytopenia, hyponatremia and nausea,^8^, ^38^ which might be related to the large number of CRM1‐regulated cargo proteins.^8^, ^9^, ^10^, ^31^ Here, we demonstrated that CRM1 expression was closely associated with cytoplasmic BATF2 expression and the prognosis of CRC patients, while CRM1 in combination with BATF2 served as a much more accurate prognostic marker. Therefore, future drugs targeting the NES region of specific tumour suppressors, such as BATF2, characterised by abnormal cytoplasmic localisation and selective tumour‐suppressing capacity, may provide higher efficacy and safety for the precise treatment of CRC.

Remarkably, we found that the OS time of CRC patients with low CRM1 and high BATF2 expression was much longer than that of patients with high CRM1 and low BATF2 expression. Thus, synergetic inhibition of CRM1 and induction of BATF2 may provide better solutions for cancer therapy. Although there are no specific BATF2 inducers at present, our previous studies demonstrated that dexamethasone (Dex), a first‐line drug in many tumours, was capable of inducing BATF2 expression.^12^, ^43^ Excitingly, two distinct clinical trials evaluated the combined effect of selinexor and Dex in patients with heavily pretreated refractory MM with limited therapeutic options, which eventually revealed a relatively high overall response rate in these patients.^43^, ^44^ Furthermore, the following IHC staining showed an increased nuclear accumulation of CRM1 cargo proteins in the bone marrow aspirates from a patient treated with selinexor and Dex, hinting at the synergetic effect of inhibiting CRM1 and inducing BATF2.^43^ Moreover, another independent clinical trial discovered that selinexor plus Dex had reduced side effects in MM patients.^45^ Therefore, it would be interesting to evaluate whether a combined therapy of selinexor plus Dex can be used in CRC patients, since selinexor monotherapy plus 5‐fluorouracil, leucovorin and oxaliplatin was not tolerable in metastatic CRC patients in a phase I clinical trial.^46^

In addition, despite of careful inquiry, we have not found an online database to investigate the associations between CRC prognosis and the subcellular localisation level of BATF2 protein, especially the cytoplasmic and nuclear BATF2 expression levels. Future proteomic studies of subcellular localisation of proteins might help solve this problem. Referring to previous reports, we found that in the study of protein degradation, although cytoplasmic TSGs were gradually degraded, they were still expressed, such as p53 and p27.^8^, ^47^ Further studies revealed cytoplasmic p53 expression as a potential biological indicator of prognosis in CRC, and it is interesting that a portion of p53 is localised to the mitochondria.^8^, ^47^, ^48^ Therefore, it would be inspiring to explore the novel functions of cytoplasmic BATF2 in future studies.

## 5 CONCLUSIONS

In summary, we firstly identified the cytoplasmic localisation of BATF2 in human CRC samples and cell lines. In vitro and in vivo studies demonstrated that BATF2 was transported out of nucleus and degraded by ubiquitin in the cytoplasm through directly binding to CRM1 via its NES region, ultimately contributing to CRC proliferation, during which process AP‐1/cyclin D1/pRb signalling pathway was involved. Furthermore, CRM1 expression was closely associated with cytoplasmic BATF2 expression and poorer CRC prognosis. Our findings demonstrate that suppressing nuclear export of BATF2 via mutating its NES region or inhibiting CRM1 expression may serve as a promising therapeutic strategy against CRC proliferation, which may shed new light on the exploration of novel combined therapeutic strategies for CRC patients.

## CONFLICT OF INTEREST STATEMENT

The authors declare no conflicts of interest.

## Supporting information

Supporting Information

Supporting Information

Supporting Information

Supporting Information

Supporting Information

Supporting Information

Supporting Information

Supporting Information

Supporting Information

Supporting Information
