# Supplementary figures and images for: Nuclear export of BATF2 enhances colorectal cancer proliferation through binding to CRM1
Source: Clin Transl Med. 2023 May 7;13(5):e1260. doi: 10.1002/ctm2.1260 (PMC10165233; doi:10.1002/ctm2.1260)

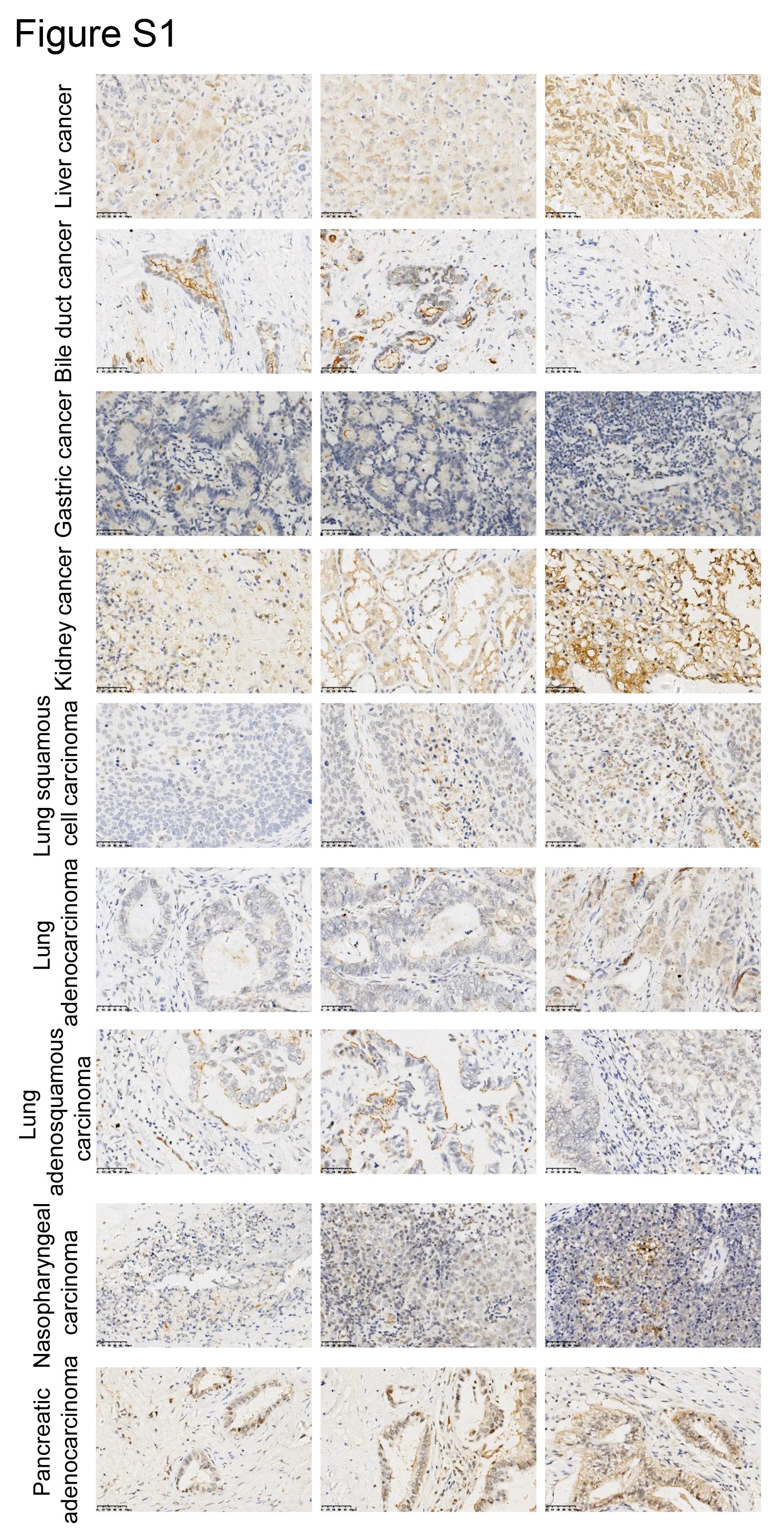

Supplement: Supplementary file 1 — Supporting Information [file CTM2-13-e1260-s003.tif]

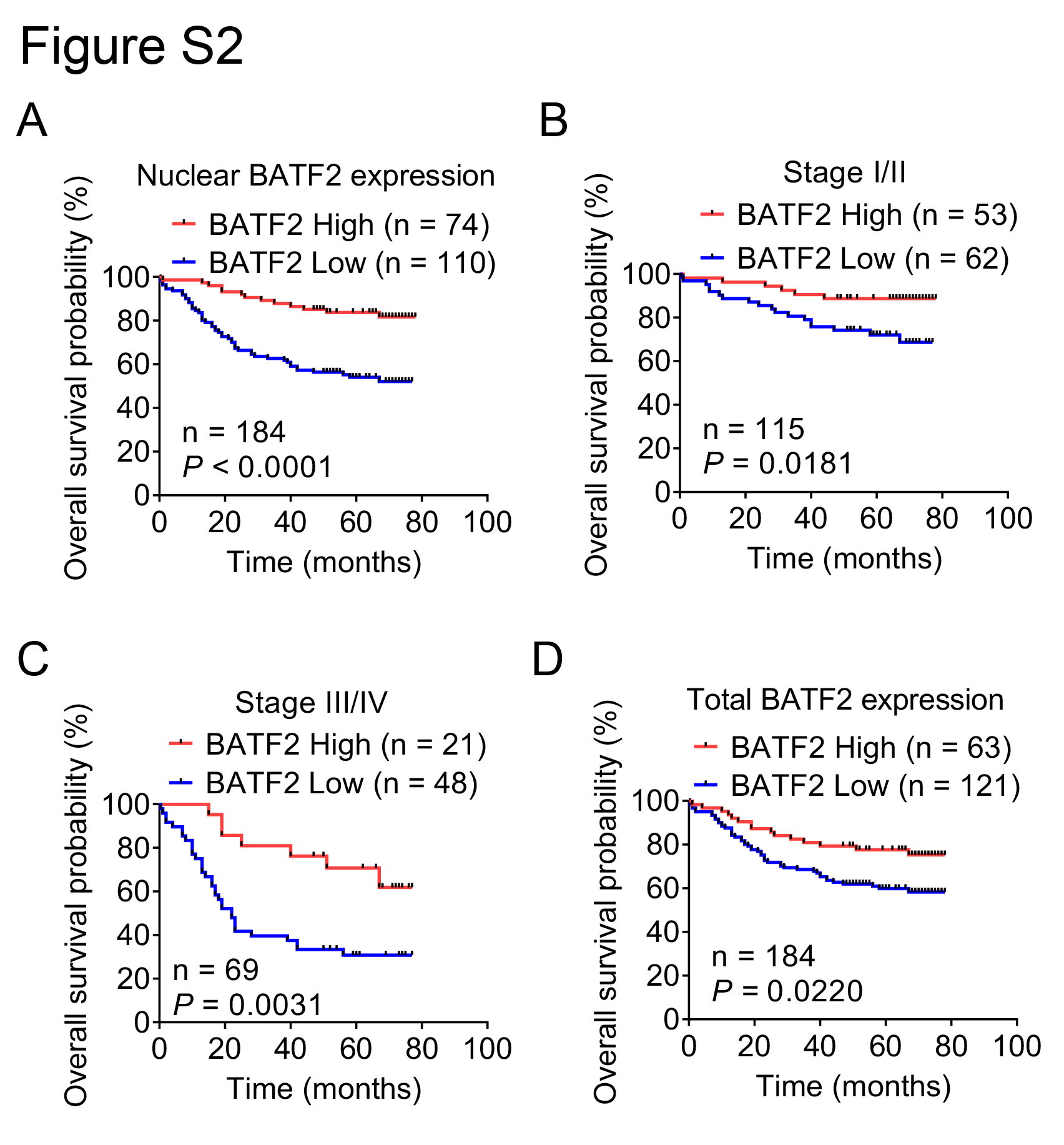

Supplement: Supplementary file 2 — Supporting Information [file CTM2-13-e1260-s002.tif]

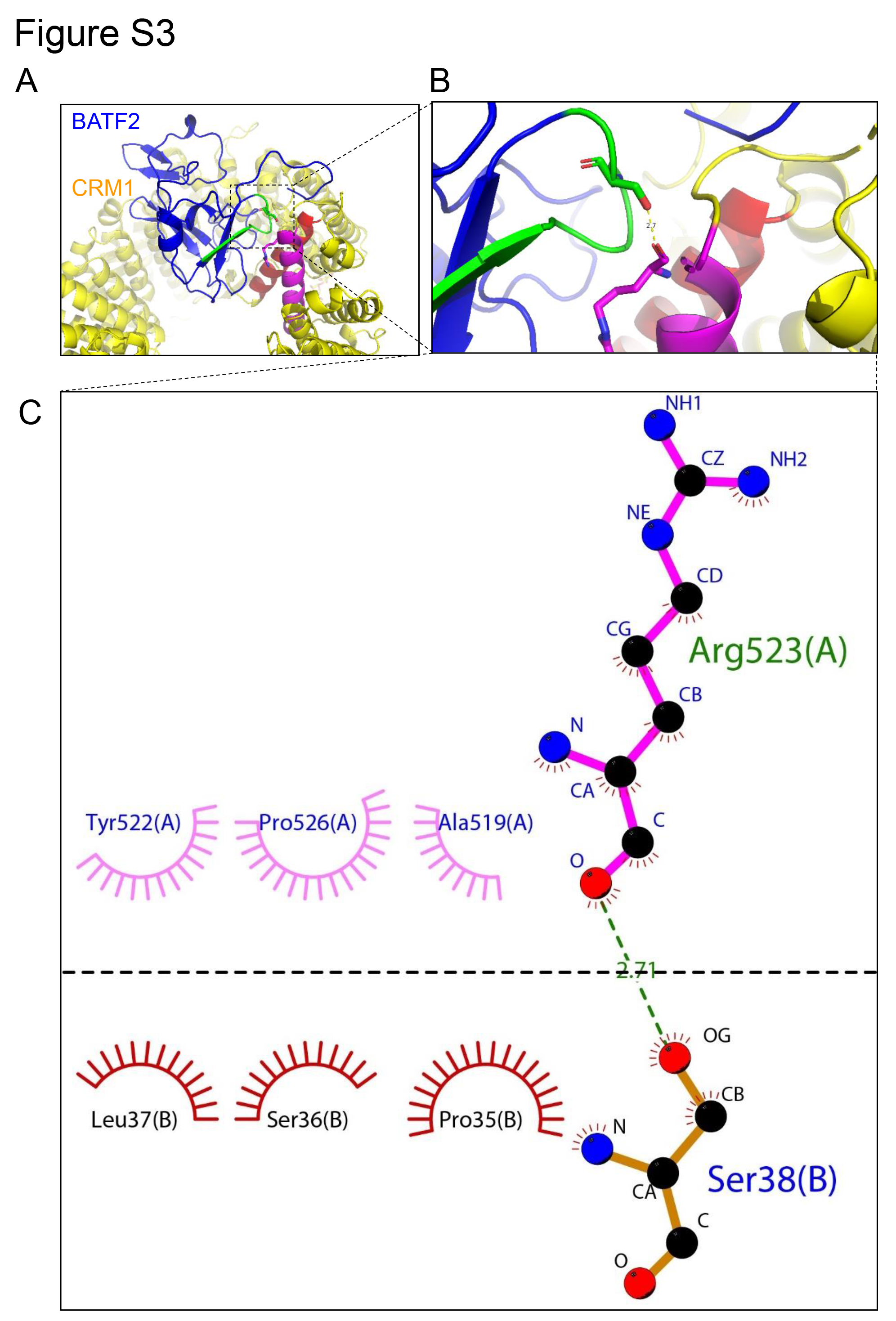

Supplement: Supplementary file 3 — Supporting Information [file CTM2-13-e1260-s008.tif]

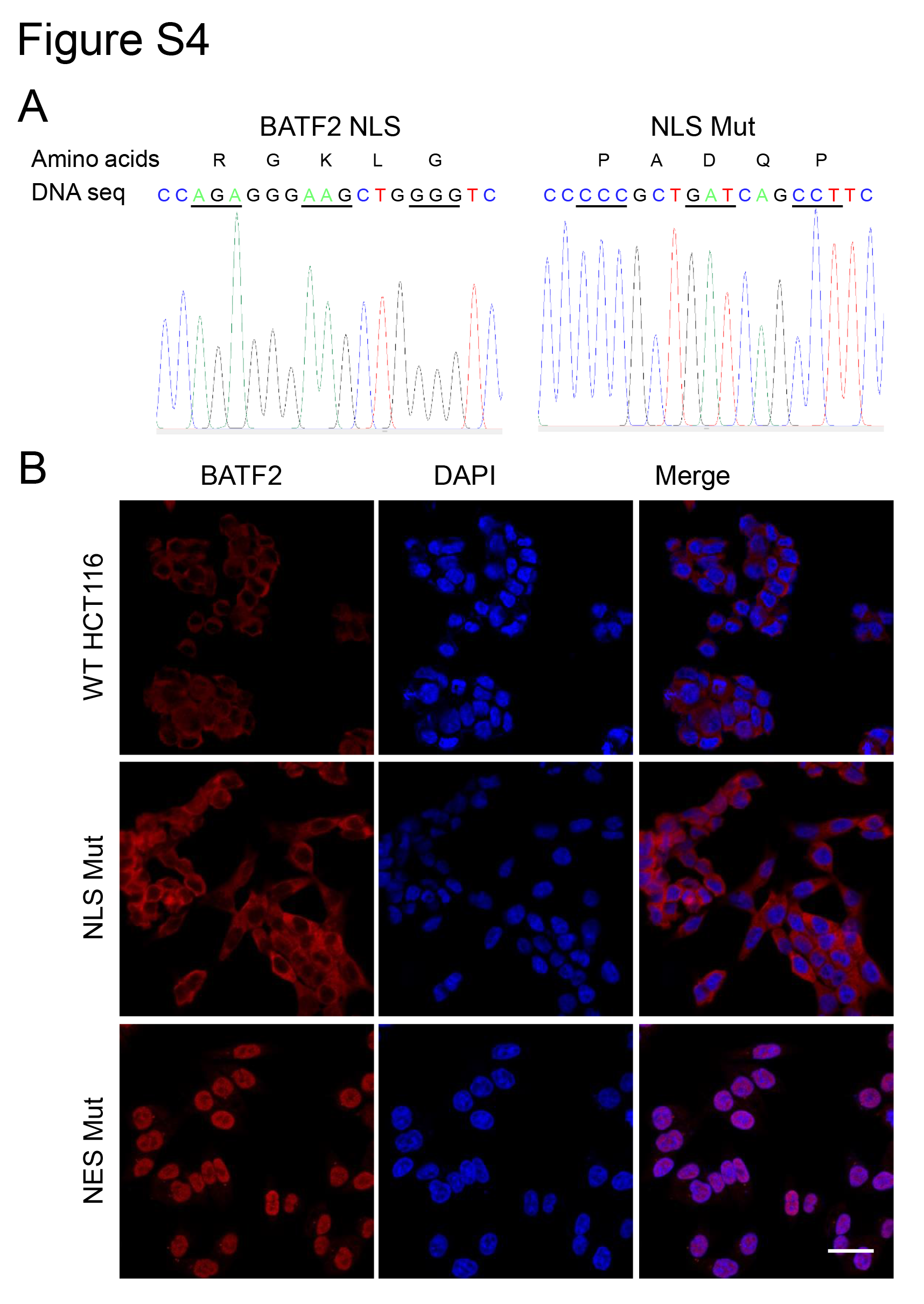

Supplement: Supplementary file 4 — Supporting Information [file CTM2-13-e1260-s006.tif]

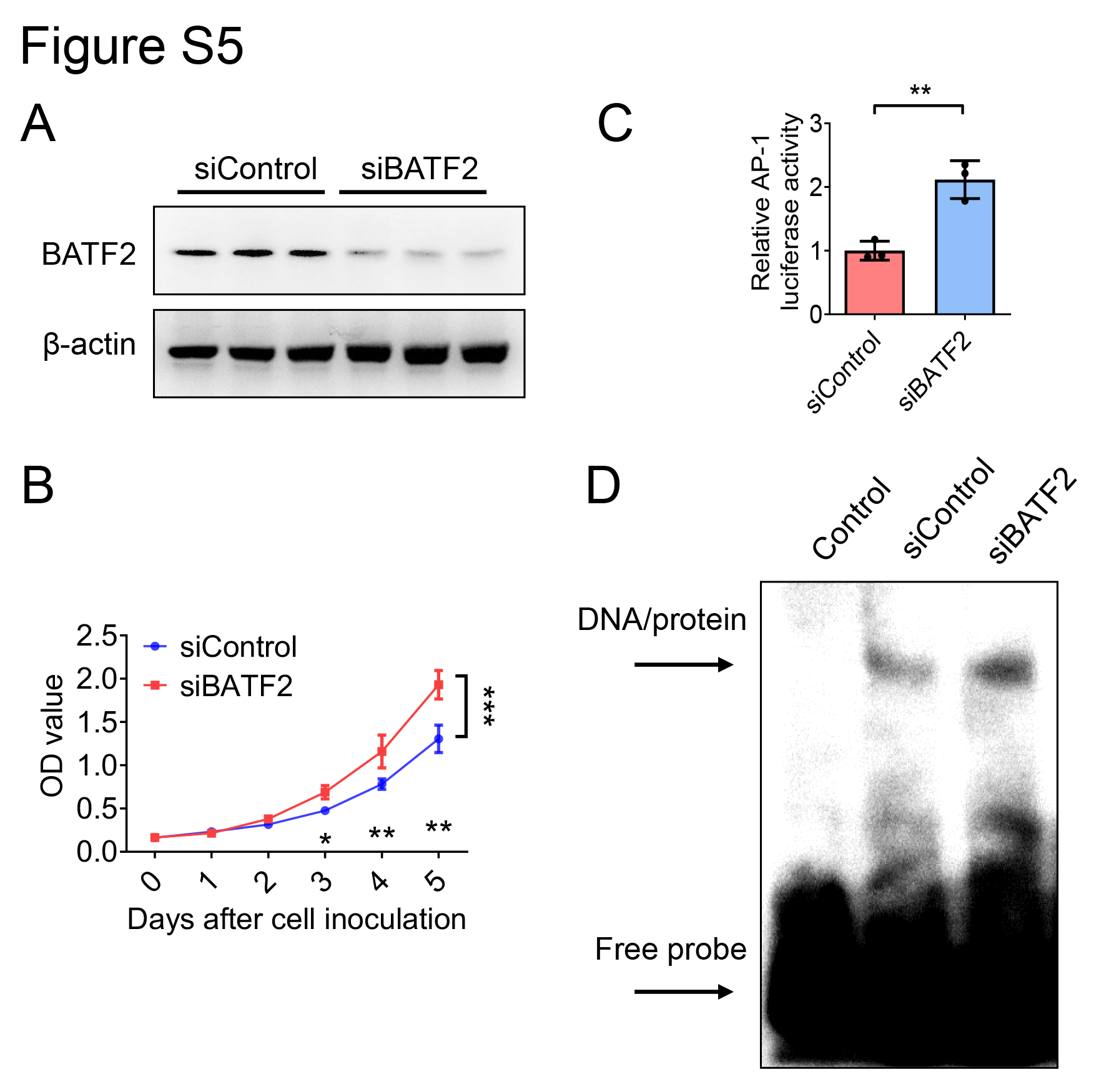

Supplement: Supplementary file 5 — Supporting Information [file CTM2-13-e1260-s005.tif]

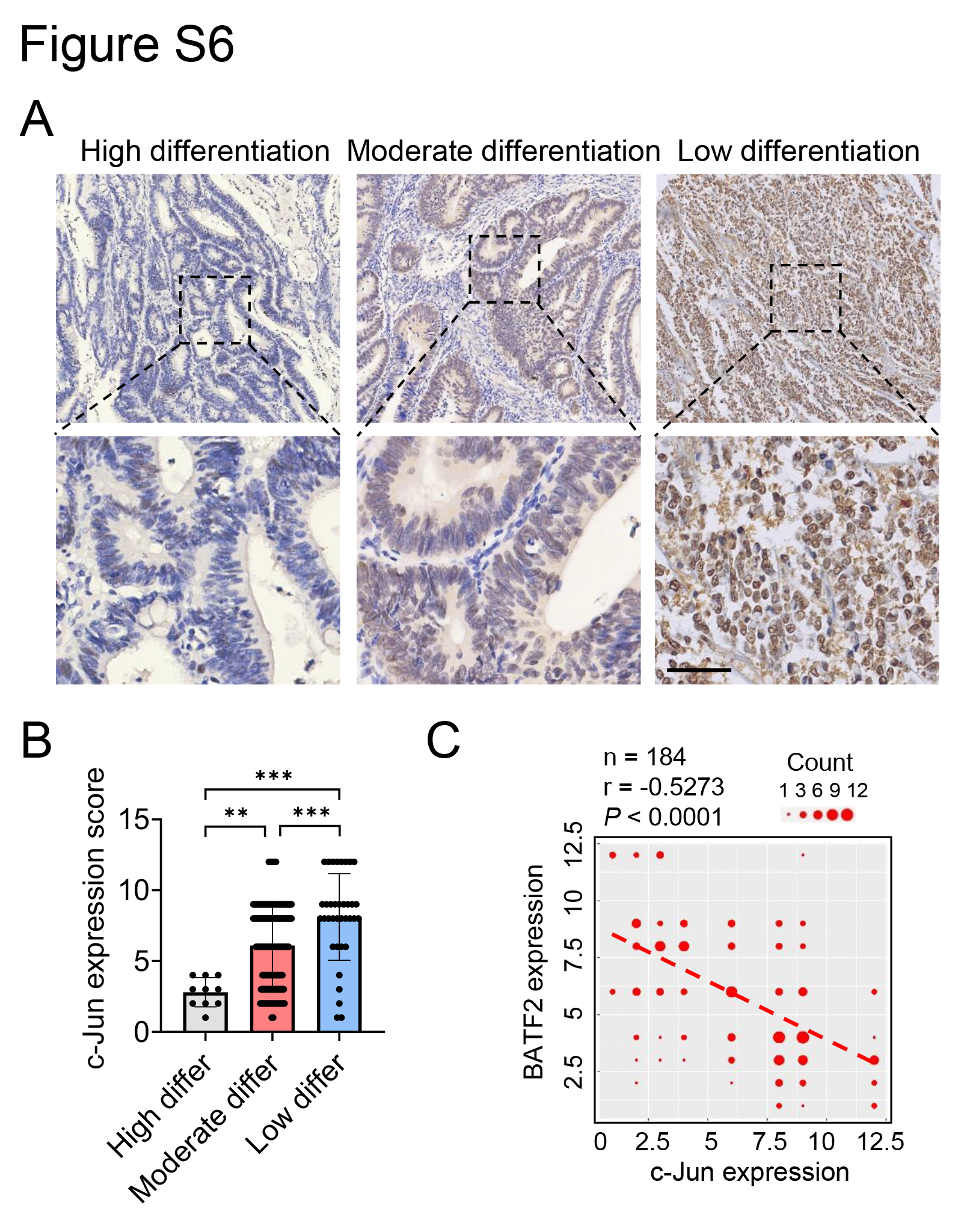

Supplement: Supplementary file 6 — Supporting Information [file CTM2-13-e1260-s009.tif]

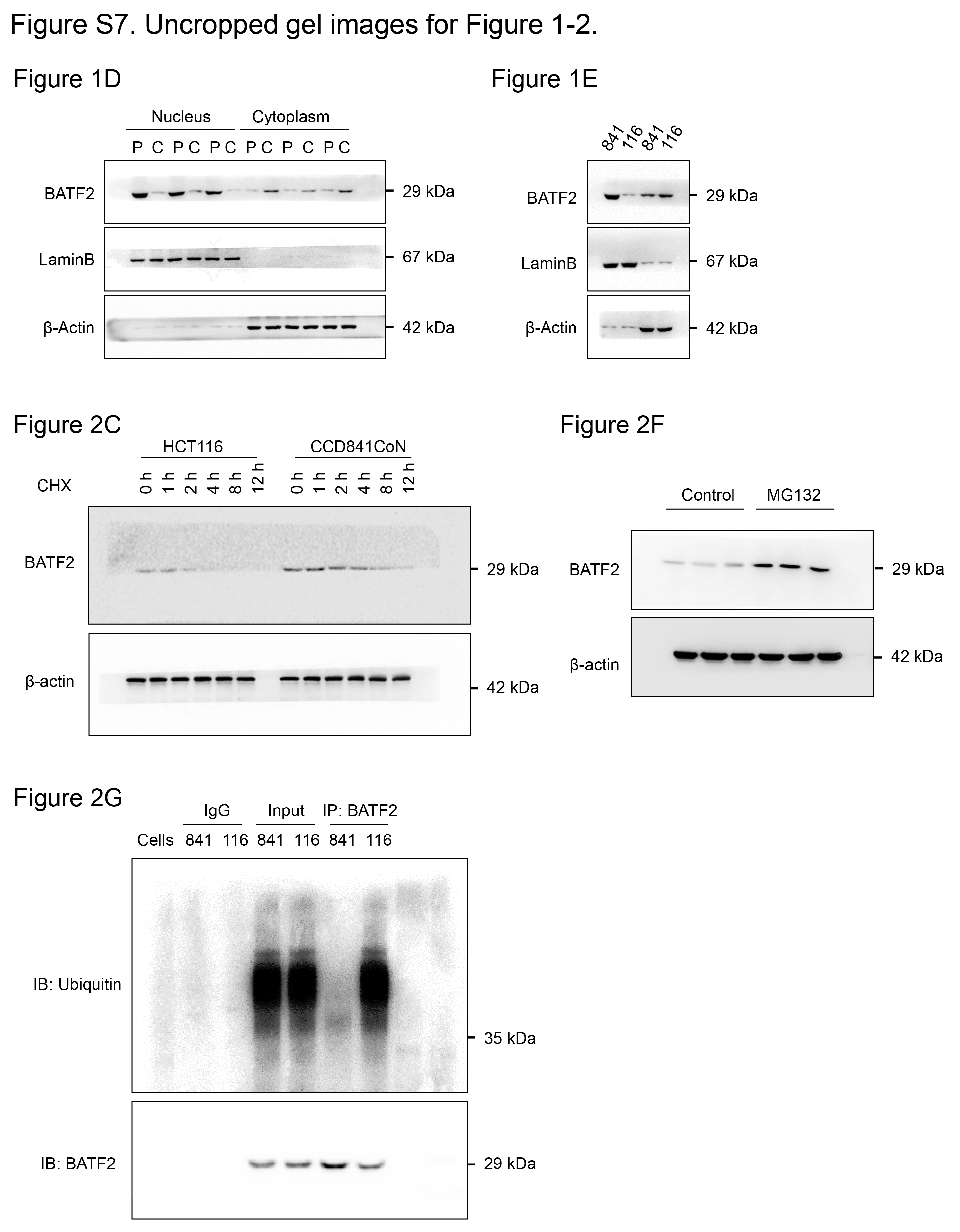

Supplement: Supplementary file 7 — Supporting Information [file CTM2-13-e1260-s010.tif]

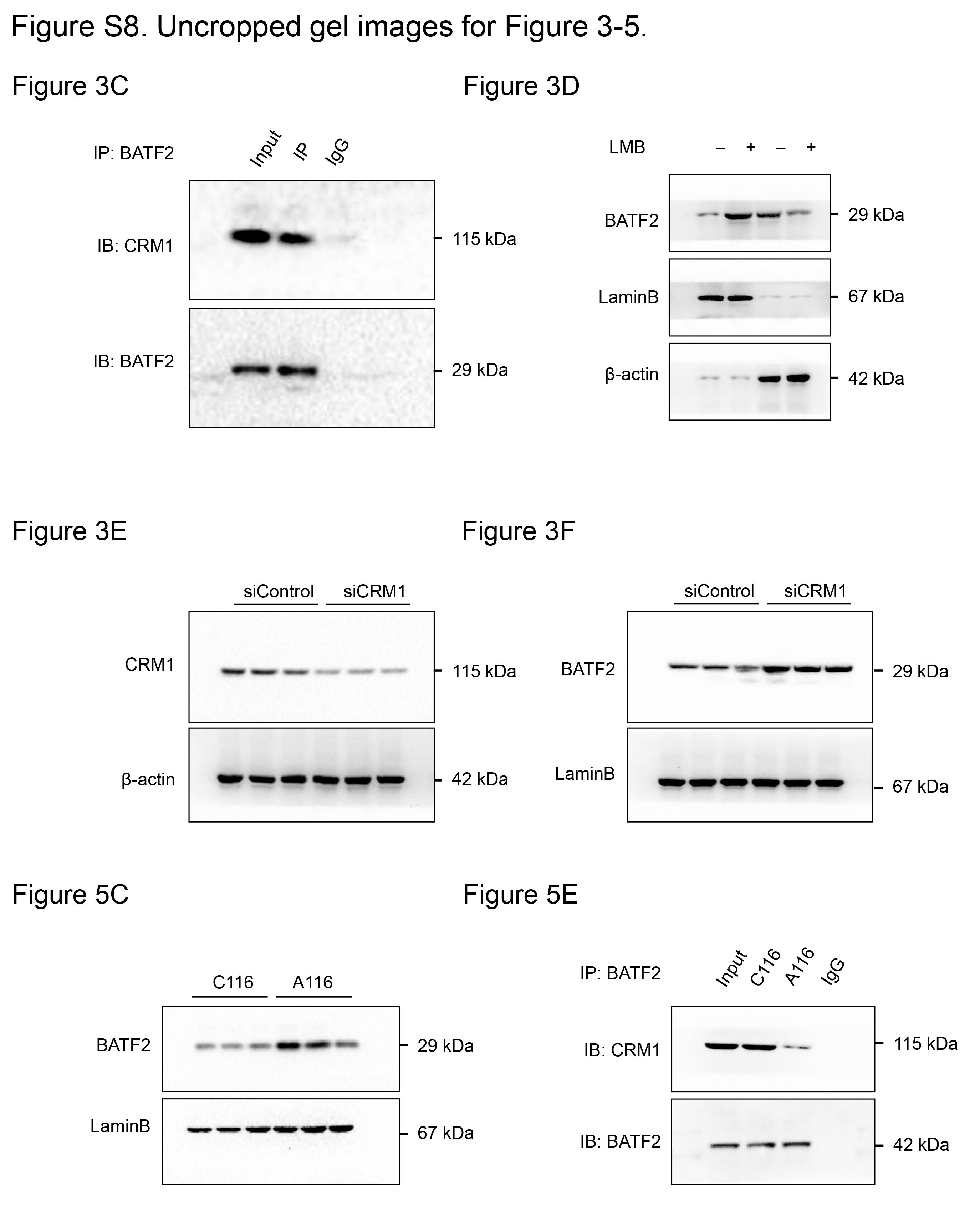

Supplement: Supplementary file 8 — Supporting Information [file CTM2-13-e1260-s007.tif]

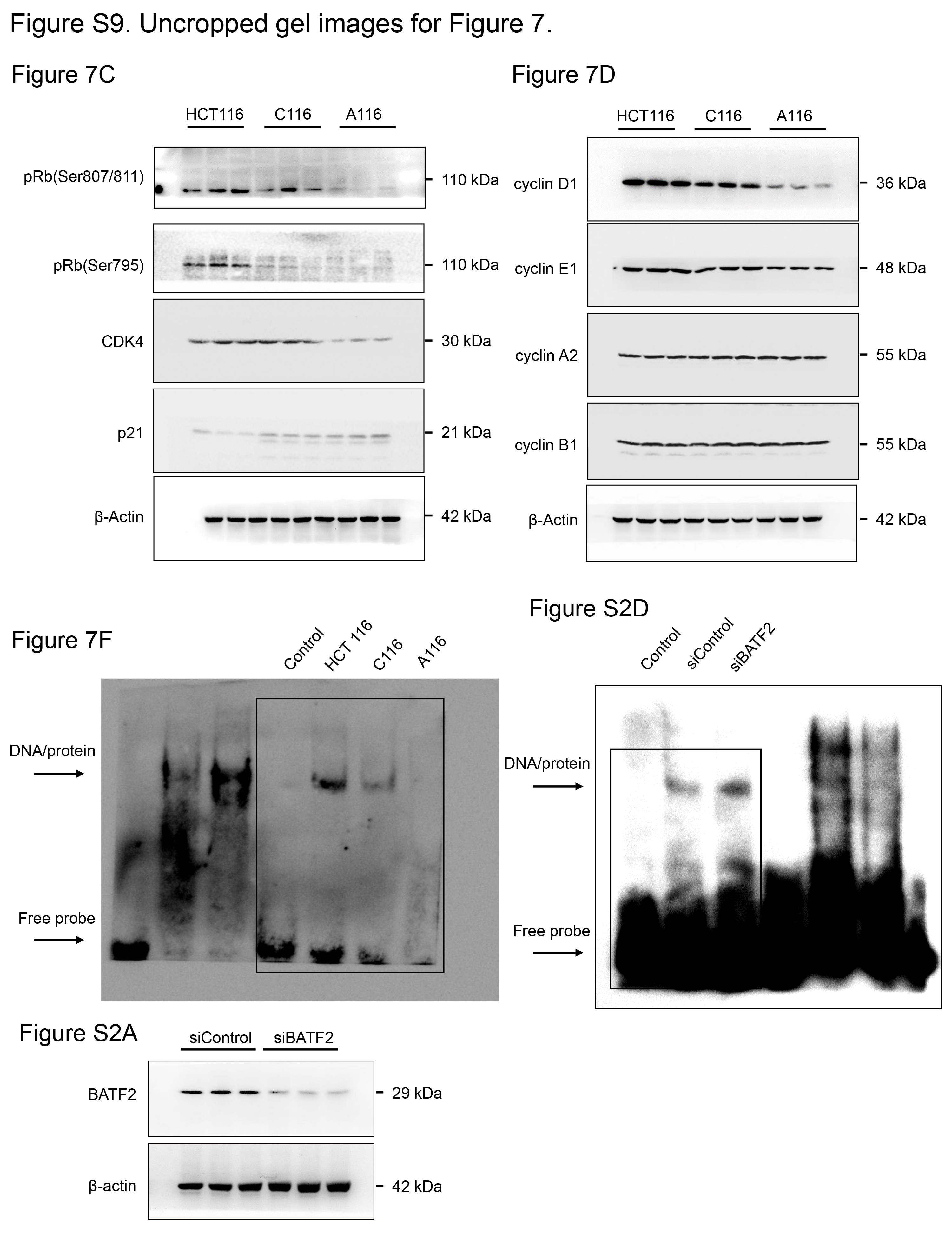

Supplement: Supplementary file 9 — Supporting Information [file CTM2-13-e1260-s004.tif]
