# Supplementary material for: Nuclear export of BATF2 enhances colorectal cancer proliferation through binding to CRM1
Source: Clin Transl Med. 2023 May 7;13(5):e1260. doi: 10.1002/ctm2.1260 (PMC10165233; doi:10.1002/ctm2.1260)
Supplement: Supplementary file 10 — Supporting Information [file CTM2-13-e1260-s001.docx]

**Supporting Information**

**Supplementary Figures Legends**

**Figure S1. Cytoplasmic localization of BATF2 was observed in multiple tumors.**

Immunohistochemical analysis of cytoplasmic localization of BATF2 in multiple tumors, including liver cancer, bile duct cancer, gastric cancer, kidney cancer, lung squamous cell carcinoma, lung adenocarcinoma, lung adenosquamous carcinoma, nasopharyngeal carcinoma and pancreatic adenocarcinoma. Scale bar: 50 μm.

**Figure S2. Higher nuclear BATF2 expression was correlated with longer survival time in CRC patients.**

(A-C) Kaplan-Meier estimates of overall survival time based on nuclear BATF2 expression levels from 184 CRC patients (*P* < 0.0001) (A), both at stage I/II (n = 115, *P* = 0.0181) (B) and stage III/IV (n = 69, *P* = 0.0031) (C). (D) Kaplan-Meier analysis of overall survival probability based on total BATF2 expression levels from 184 CRC patients (*P* = 0.0220).

**Figure S3. Bioinformatic analysis predicts an interaction between BATF2 and CRM1.**

(A) Bioinformatic prediction of the binding between BATF2 and CRM1 by using ZDOCK software. The NES region of BATF2 in the complex model is highlighted with green color. (B-C) The interaction network diagram was performed by using ligplot software. In the result of docking, BATF2 and CRM1 may form the hydrogen bond and interact through the hydrogen bond. ARG523 is a residue of CRM1 protein, while Ser38 stands for Ser157 in the BATF2 peptide. Other residues produce hydrophobic or van der Waals forces.

**Figure S4. NLS region mediates the translocation of BATF2 from cytoplasm to nucleus.**

(A) The NLS (RGKLG) of BATF2 was mutated into (PADQP), which were demonstrated by DNA sequencing. (B) Vector control or recombinant BATF2 plasmids with mutated NLS region (NLS Mut) or mutated NES region (NES Mut) were separately transfected into HCT116 cells by using Lipofectine2000, and then cells were harvested for immunofluorescence analysis of BATF2 expression. Scale bar: 10 μm.

**Figure S5. Knockdown of BATF2 expression enhanced CRC cell growth.**

(A) Western blot analysis of BATF2 expression in HCT116 cells transfected with scramble siRNAs (siControl) or siRNAs against BATF2 (siBATF2). (B) The cell proliferation of HCT116 cells transfected with siControl or siBATF2 at the indicated time was determined by using CCK-8 assay. (C) pAP-1-Luc was co-transfected with β-gal vector into cells in (A) using Lipofectamine 2000 for luciferase reporter gene assay, which was normalized against β-gal activity. (D) EMSA analysis of the DNA binding activity of AP-1 in cells in (A). Data are expressed as mean ± SD. **P <* 0.05, ***P* < 0.01, ****P* < 0.001.

**Figure S6. Nuclear BATF2 expression was negatively associated with AP-1 in CRC samples.**

(A) Representative IHC staining of c-Jun, the AP-1 major subunit, in CRC samples with different differentiation. (B) c-Jun expression levels were analyzed by using ANOVA analysis among highly, moderately and lowly differentiated CRC tissues. (C) The negative correlation between the protein levels of BATF2 and CRM1 by using Graphpad Prism 8.0 software. The scatter diagram was drawn by using the ggplot2 program package of R language, with the number of repeated dots represented by Count. Data are expressed as mean ± SD. ***P <* 0.01, ****P* < 0.001.

**Supplementary Table**

Table S1. Associations between BATF2 levels and clinicopathological parameters of CRC patients

| Parameters | Cytoplasmic staining | | *P* value | Nuclear staining | | *P* value |
| --- | --- | --- | --- | --- | --- | --- |
|  | High | Low |  | High | Low |  |
| **Cases** | 67 | 117 |  | 74 | 110 |  |
| **Gender** |  |  | 0.513 |  |  | 0.673 |
| Female | 31 | 60 |  | 38 | 53 |  |
| Male | 36 | 57 |  | 36 | 57 |  |
| **Age (years)** |  |  | 0.363 |  |  | 0.938 |
| ≤ 60 | 28 | 41 |  | 28 | 41 |  |
| > 60 | 39 | 76 |  | 46 | 69 |  |
| **Diagnosis** |  |  | 0.565 |  |  | 0.172 |
| Adenocarcinoma | 49 | 90 |  | 52 | 87 |  |
| Mucinous adenocarcinoma | 18 | 27 |  | 22 | 23 |  |
| **Classification** |  |  | 0.567 |  |  | 0.071 |
| Ulcer | 28 | 54 |  | 27 | 55 |  |
| Mass | 39 | 63 |  | 47 | 55 |  |
